# Supplementary figures and images for: The E3 ubiquitin ligase Cul4b promotes CD4+ T cell expansion by aiding the repair of damaged DNA
Source: PLoS Biol. 2021 Feb 1;19(2):e3001041. doi: 10.1371/journal.pbio.3001041 (PMC7888682; doi:10.1371/journal.pbio.3001041)

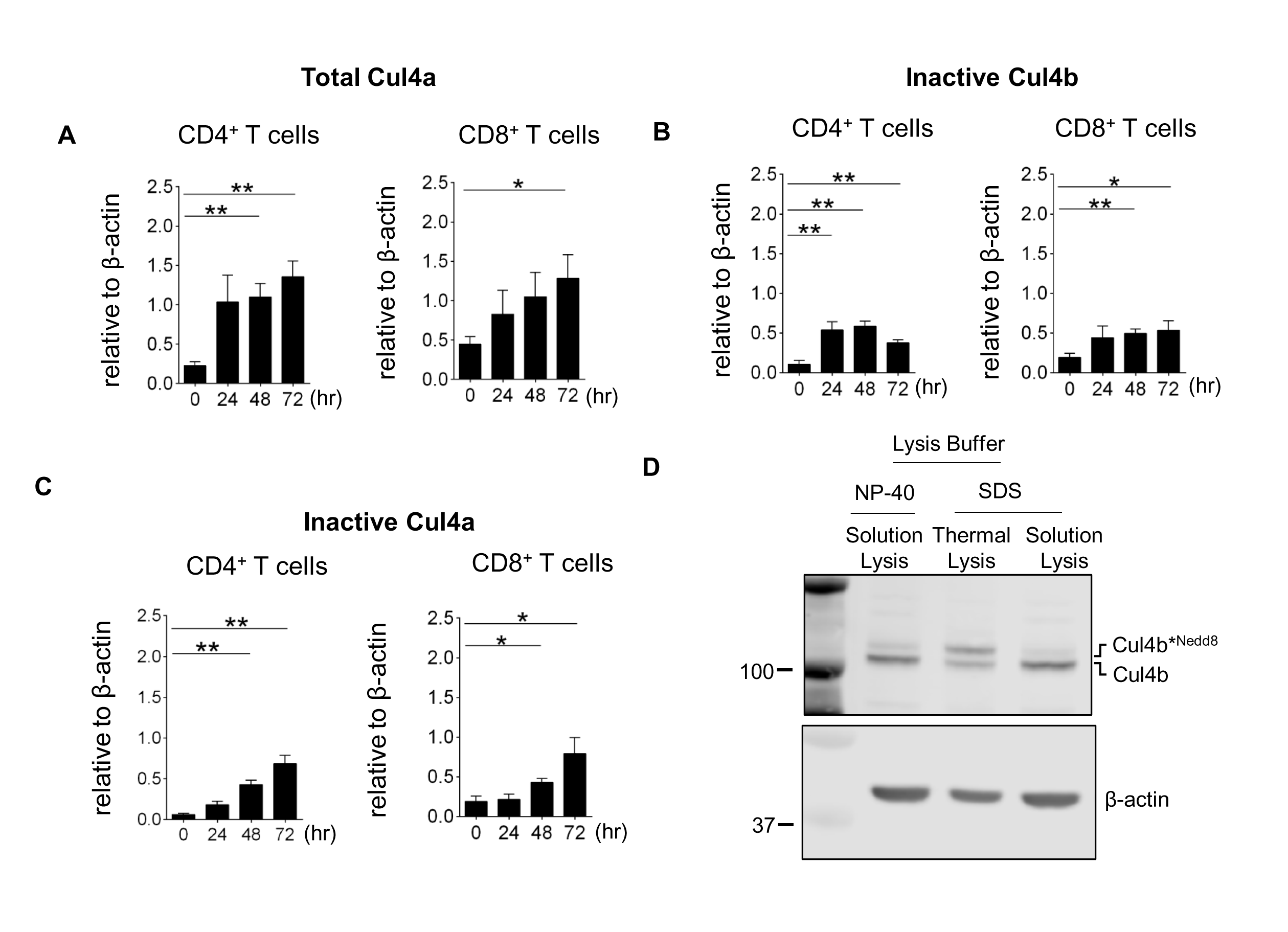

Supplement: S1 Fig — Naive CD4+ T and CD8+ T cells from control mice (C57BL/6 mice) were stimulated with anti-CD3 and anti-CD28 mAbs. Expression of Cul4a and Cul4b was monitored by immunoblotting. (A–C) The quantitative data of 4 independent experiments are shown. (B) shows inactive (nonneddylated) Cul4b in CD4+ and CD8+ T cells at different time points. (C) shows inactive Cul4a in CD4+ and CD8+ T cells. Data were quantitated using Image J software and are represented as mean ± SEM (*P < 0.05 **P < 0.01, ***P < 0.001 by Student t test; ns, not significant, P > 0.05 by Student t test). (D) The activated CD4+ T cells were lysed using different conditions as described above, and presence of neddylated and nonneddylated forms of the protein were determined by immunoblotting. For numerical raw data, please see S1 Data. Cul4a, Cullin-4a; Cul4b, Cullin-4b; mAbs, monoclonal antibodies; SEM, standard error of mean. (TIF) [file pbio.3001041.s001.tif]

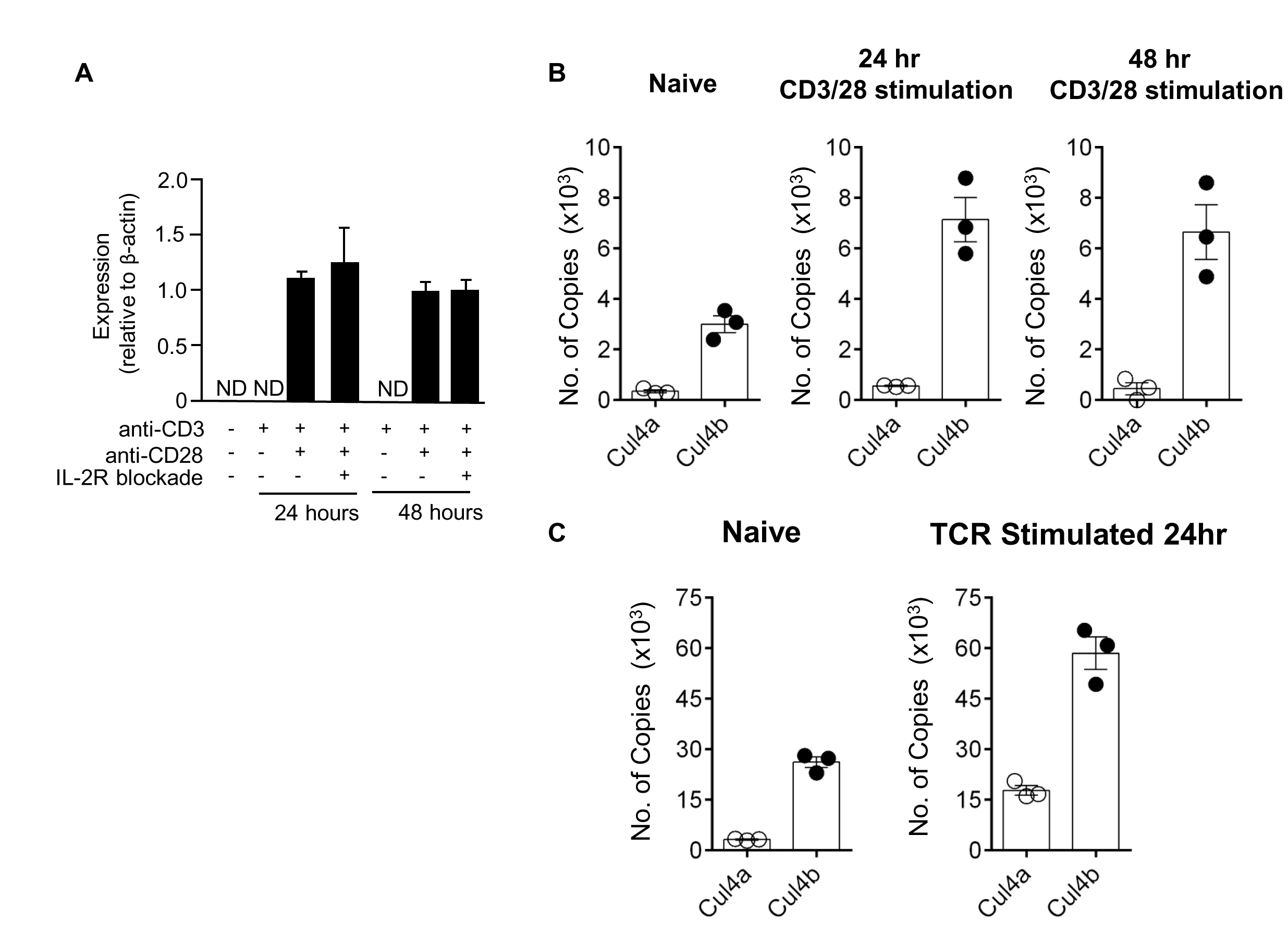

Supplement: S2 Fig — (A) Naive CD4+ T cells from control mice were activated either with anti-CD3 mAb or anti-CD3 and anti-CD28 mAbs. In case of anti-CD3/CD28 mAb stimulation, cells were either neutralized for IL-2R by adding anti-IL-2R antibody (10 μg/ml) or left as such. The mRNA expression of Cul4b was detected by RT-PCR. The Cul4b expression was determined in naive and activated CD4+ T cells, and β-actin was used as internal control. Data are represented as mean ± SEM of 3 independent experiments. (B) Quantification of protein abundance of Cul4a and Cul4b in CD4+ T cells using mass spectrometry. The copy numbers of Cul4a and Cul4b in naïve and TCR-activated (24 and 48 h) CD4+ T cells are shown and were calculated using the proteomic ruler method. (C) The bar graph shows the copy numbers of Cul4a and Cul4b in naïve and antigen-stimulated CD4+ T cells; the data were analyzed from the data set reported by Howden and colleagues [38]. For numerical raw data, please see S2 Data. Cul4b, Cullin-4b; IL-2R, IL-2 receptor; mAbs, monoclonal antibodies; RT-PCR, real-time PCR; SEM, standard error of mean; TCR, T cell receptor. (TIF) [file pbio.3001041.s002.tif]

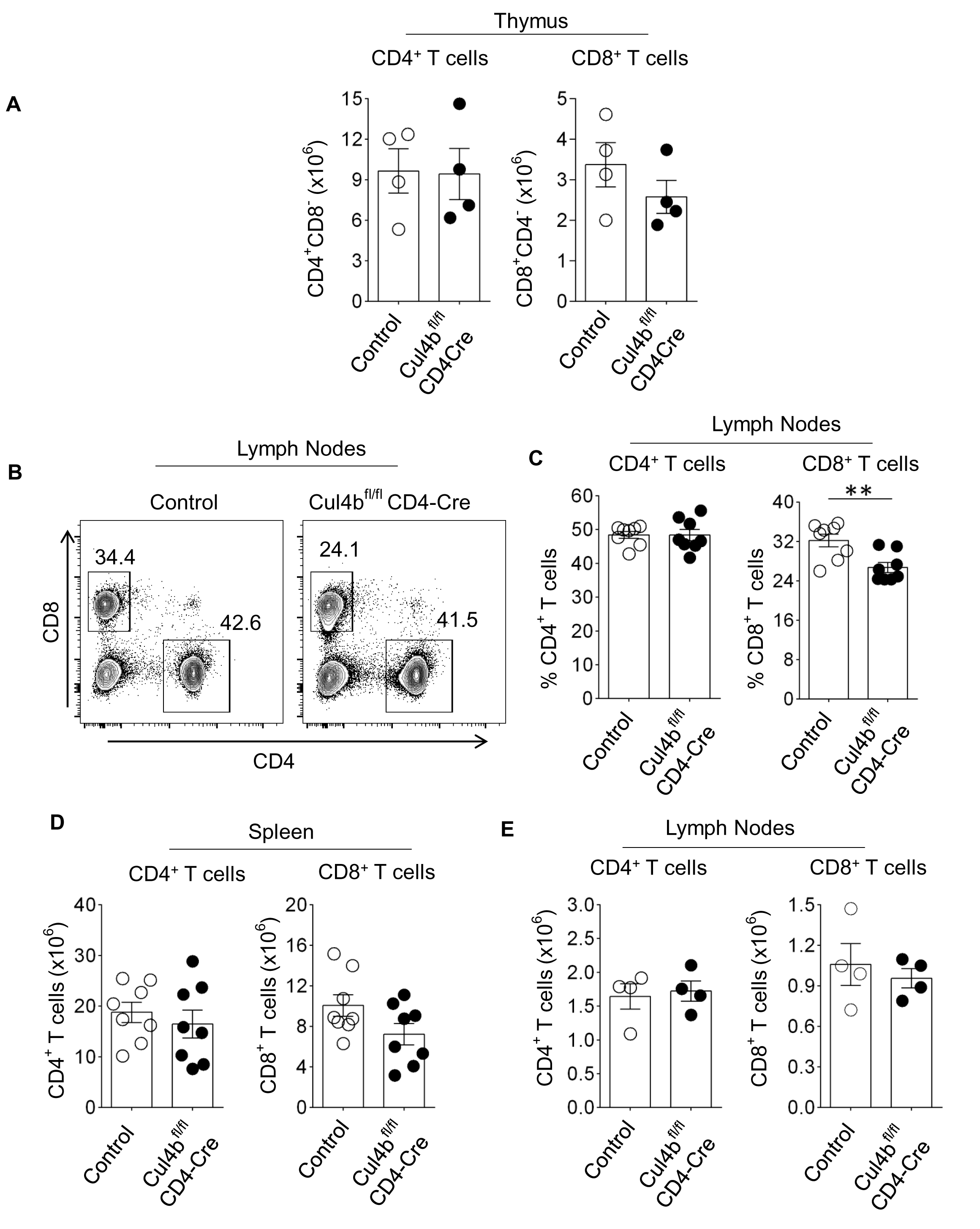

Supplement: S3 Fig — (A) The numbers of various T-cell populations in the thymus of control (Cul4bfl/fl) and Cul4bfl/fl-CD4Cre mice. The bar graphs show the mean ± SEM of 4 sets of mice (ns, not significant, P > 0.05 by Student t test). (B and C) The percentages of various T-cell populations in the lymph of control (Cul4bfl/fl) and Cul4bfl/fl-CD4Cre mice were assessed by flow cytometry. The bar graphs show the mean ± SEM of 8 sets of mice (**P < 0.01 by Student t test; ns, not significant, P > 0.05 by Student t test). The mice were paired with respective to age, gender, cage, and time of takedown. (D and E) The numbers of various T-cell populations in the spleen and lymph nodes of control (Cul4bfl/fl) and Cul4bfl/fl-CD4Cre mice are shown. The bar graphs show the mean ± SEM of 4 sets of mice (ns, not significant, P > 0.05 by Student t test). For numerical raw data, please see S3 Data. Cul4b, Cullin-4b; SEM, standard error of mean. (TIF) [file pbio.3001041.s003.tif]

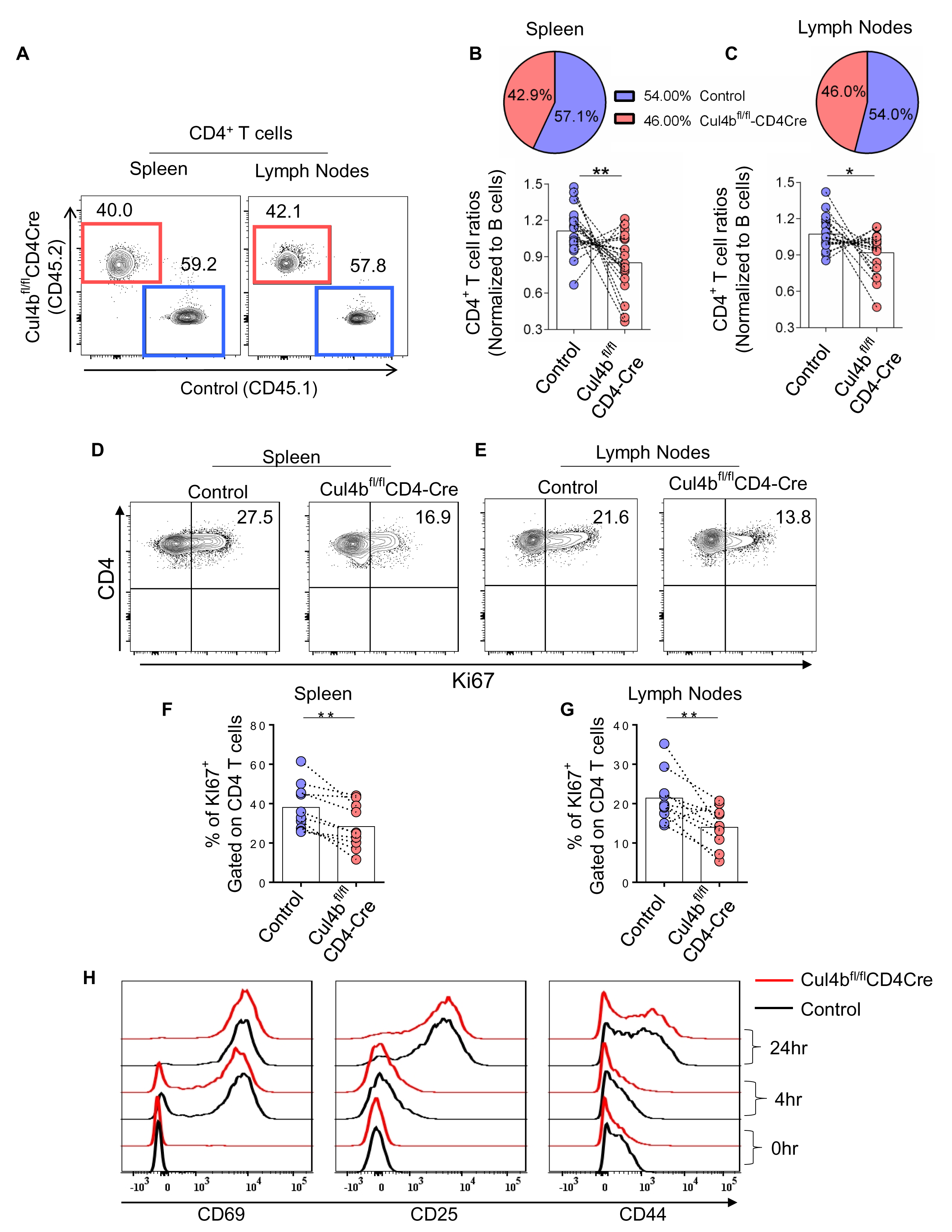

Supplement: S4 Fig — (A) The comparison of the total CD4+ and B cell populations in the spleen and lymph nodes of irradiated recipient chimeric mice after reconstitution of bone marrow cells from control mice (CD45.1+) and Cul4bfl/fl-CD4Cre mice (CD45.2+). (B and C) The line graphs show the relative ratios of CD4+ T cells in spleen and lymph nodes. Ratios were calculated by dividing the percentages of CD4+ T cells of each genotype with the percentages of B cells from the same genotype. The pie chart depicts the relative percentages of the control and Cul4bfl/fl-CD4Cre CD4+ T cells. (D–G) CD4+ T cells in spleen and lymph nodes were analyzed for Ki67 expression, a marker for proliferation. Representative plots show the frequencies of Ki67-positive control (CD45.1+) and Cul4bfl/fl-CD4Cre (CD45.2+) CD4+ T cells. The line graphs show the relative frequencies of Ki67+CD4+ T cells in spleen and lymph nodes. (H) The expression of activation markers CD69, CD25, and CD44 on control and Cul4bfl/fl-CD4Cre CD4+ T cells was analyzed by flow cytometry. The naïve CD4+ T cells were stimulated with anti-CD3/CD28 mAb for 4 h and 24 h cells. The expression in naive and stimulated (4 and 24 h) control and Cul4bfl/fl-CD4Cre CD4+ T cells is shown. Black lines represent the control and red lines represent the Cul4bfl/fl-CD4Cre CD4+ T cells. For numerical raw data, please see S5 Data. Cul4b, Cullin-4b; mAb, monoclonal antibody. (TIF) [file pbio.3001041.s004.tif]

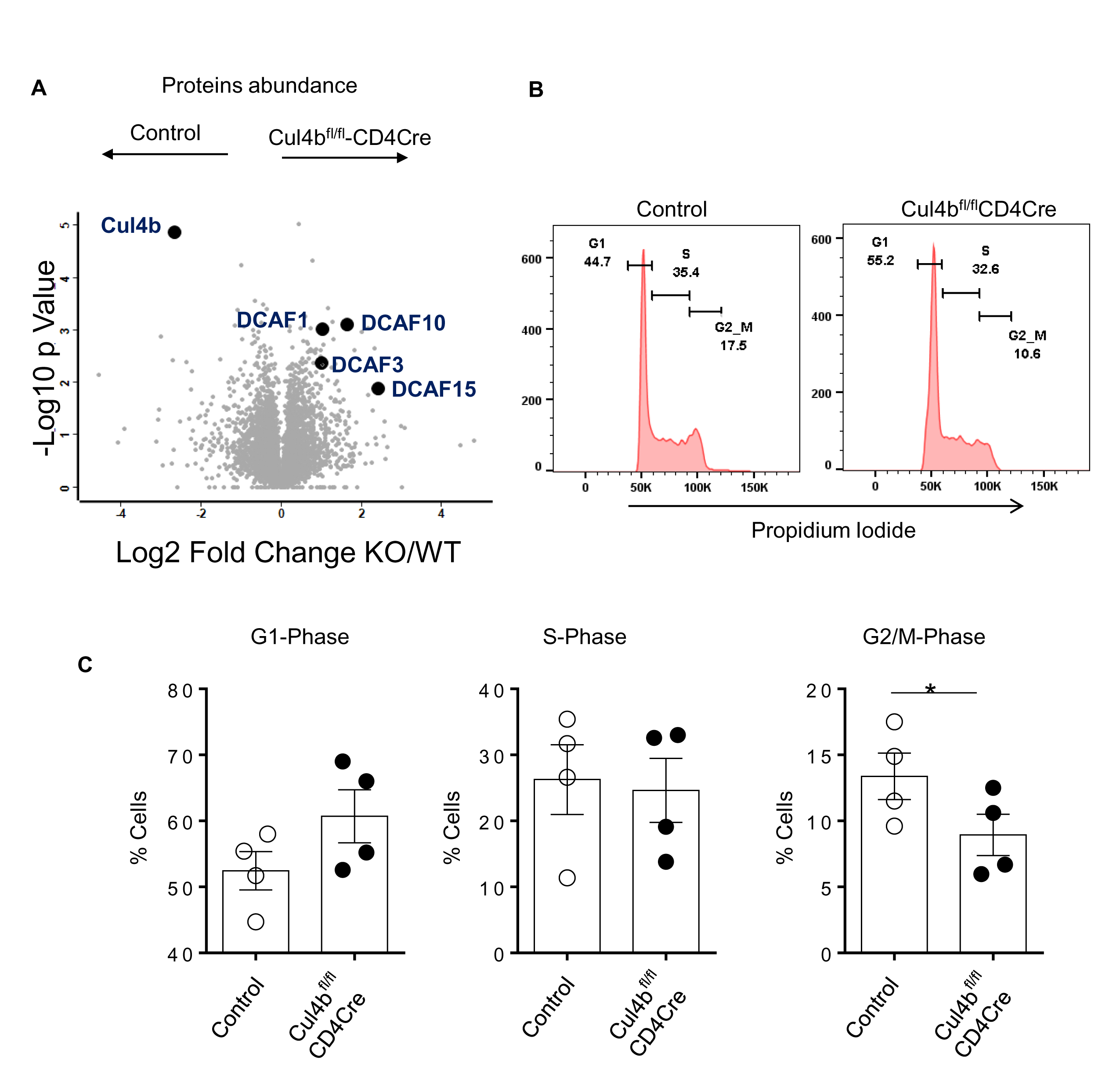

Supplement: S5 Fig — (A) Cul4b-deleted (Cul4bfl/fl-CD4Cre) and control (Cul4bfl/fl) CD4+ T cells were cultured for 3 days and then rested in IL-2 for 2 days. After resting, these cells were restimulated for 4 h with anti-CD3/CD28 mAbs (5 μg/ml). Proteins were quantified by iBAQ intensities values and were compared between control and Cul4b-deleted CD4+ T cells to generate fold changes. Volcano plot shows the differentially regulated proteins; blue dots indicate 517 proteins that were different (P < 0.05, n = 3). Proteins with higher abundance in CD4+ T cells derived from Cul4bfl/fl-CD4Cre are on the right side of the plot and those in control CD4+ T cells are on the left side. (B) CD4+ T cells were purified from control and Cul4bfl/fl-CD4Cre mice and stimulated with anti-CD3 and ant-CD28 (5 μg/ml) for 72 h. Cells were alcohol fixed and permeabilized and stained with PI, and DNA content was analyzed by flow cytometry. Doublets and dead cells were excluded. The histograms represent PI fluorescence intensity of CD4+ T cells. (C) The histogram shows the percentage of cells in G1, S, and G2/M phases of the cell cycle. Data are representative of 4 independent experiments. (*P < 0.05, by Student t test). For numerical raw data, please see S6 Data. Cul4b, Cullin-4b; iBAQ, intensity-based absolute quantification; IL-2, interleukin 2; mAbs, monoclonal antibodies; PI, propidium iodide. (TIF) [file pbio.3001041.s005.tif]

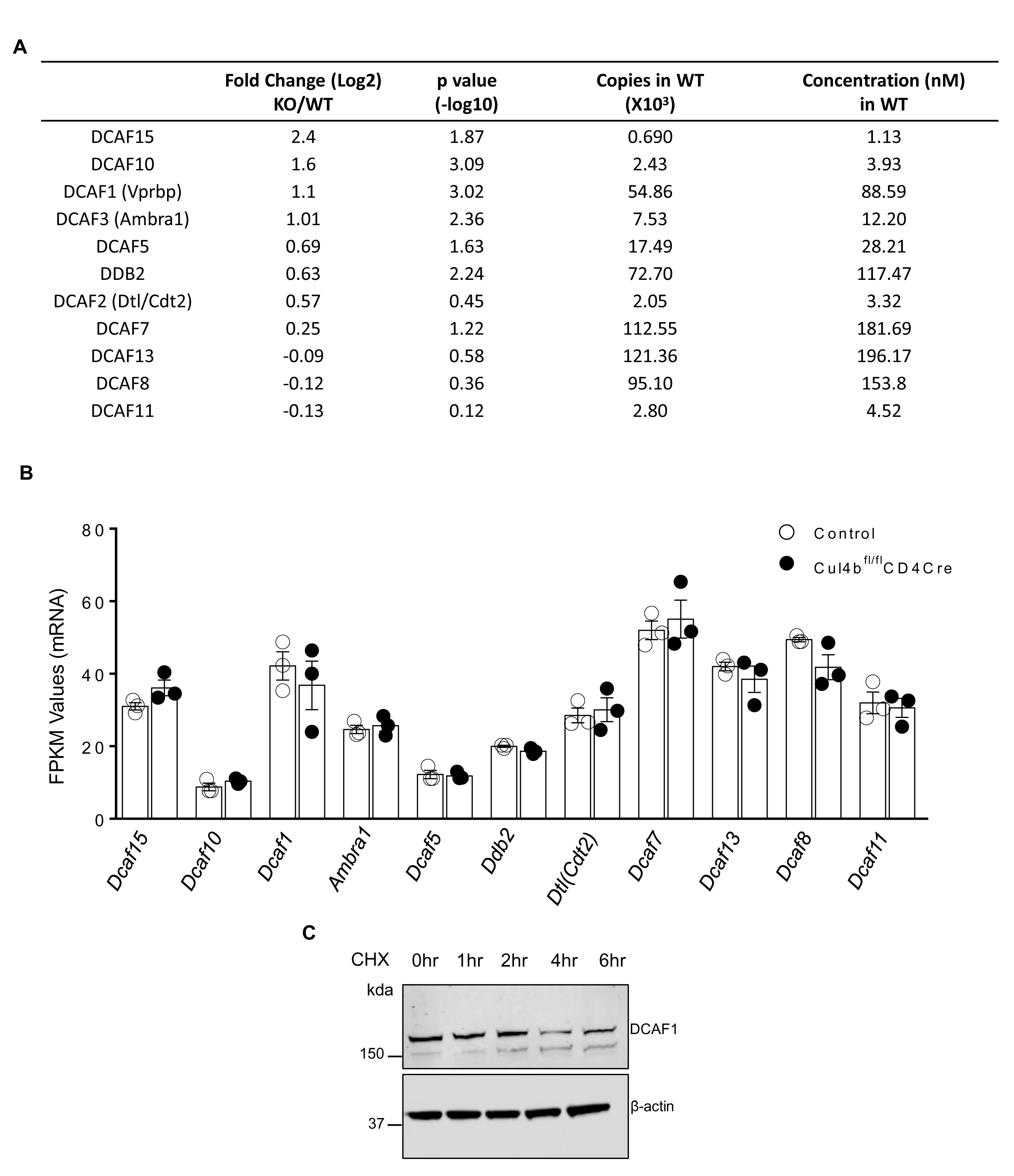

Supplement: S6 Fig — (A) The list of known substrate receptors, their fold changes of protein abundance in Cul4bfl/fl-CD4Cre (KO) to control cells (WT), P value, their respective copies, and concentration in control (WT) CD4+ T cells is shown. DCAF15, 10, 3, and 1 have log2 fold change of greater that 1 (= 2-fold change); among these DCAF1 has higher copy number and concentration. (B) Comparison of the transcript levels (by FPKM values) of substrate receptors listed in (A) in control (WT) and Cul4bfl/fl-CD4Cre CD4+ T cells. (C) The protein turnover of DCAF1 in anti-CD3 and anti-CD28 activated (for 24 h) control CD4+ T cells in the presence of translation inhibitor CHX was determined by immunoblotting; β-actin was used as loading control. For numerical raw data, please see S7 Data. For supporting data set, please see S1 and S2 Tables. CHX, cycloheximide; KO, knockout; WT, wild-type. (TIF) [file pbio.3001041.s006.tif]

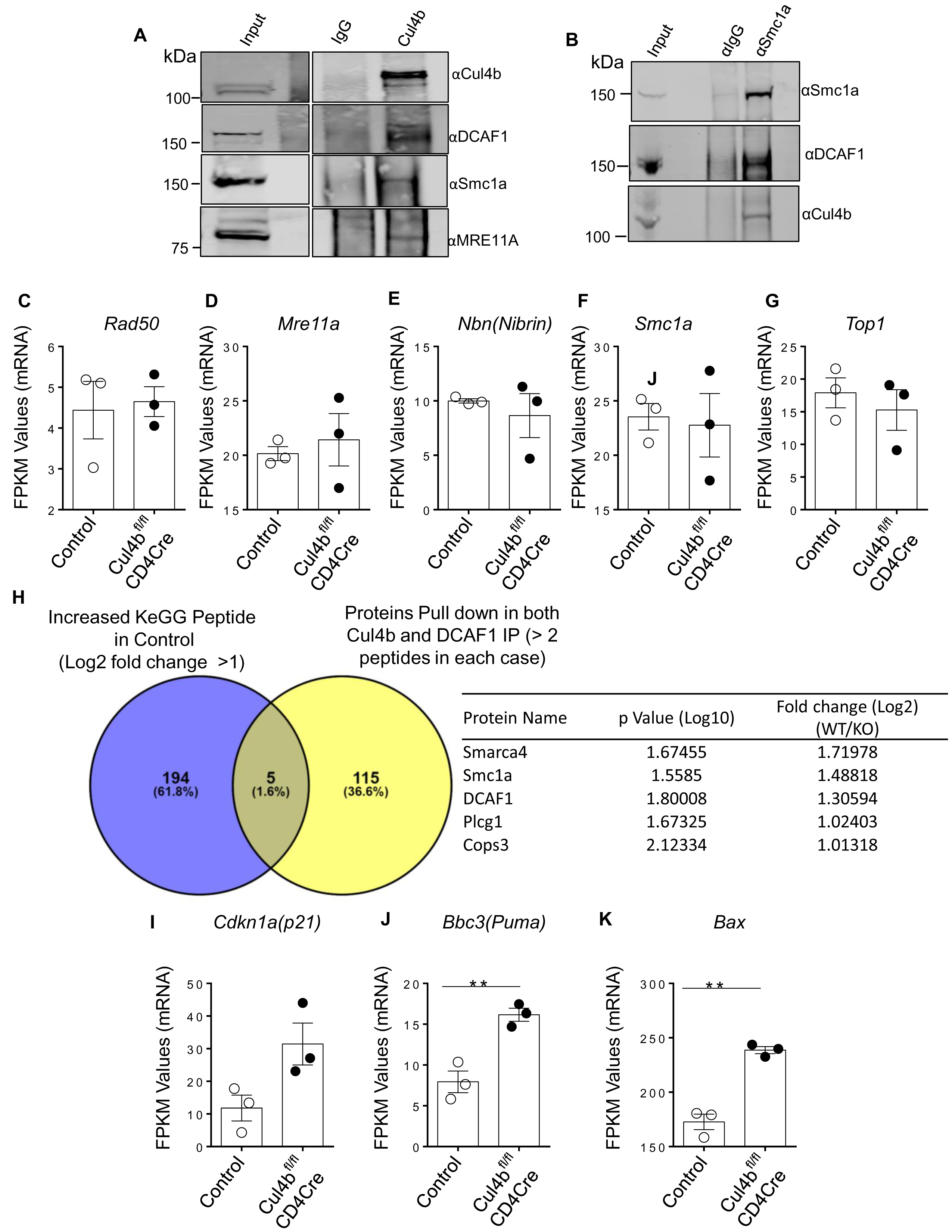

Supplement: S7 Fig — (A) Coimmunoprecipitation of DCAF1, MRE11A, and SMC1A with Cul4b in TCR-stimulated CD4+ T cells. Protein lysates were preincubated with DNase I (10 μg/ml) for 30 min on ice prior to immunoprecipitation. (B) Coimmunoprecipitation of DCAF1 and Cul4b with SMC1A in TCR-stimulated CD4+ T cells. (C–G) Comparison of the transcript levels (by FPKM values) of proteins identified to be interacting with both Cul4b and DCAF1 in control (WT) and Cul4bfl/fl-CD4Cre CD4+ T cells. (H) Venn diagram shows the overlap of the proteins with increased diglycine-modified peptides (K-ε-GG) in control T cells and proteins identified as common interacting partners of Cul4b and DCAF1. Diglycine-modified peptides with 2-fold differences and proteins with at least 2 peptides IP’ed in mass spectrometry were used. (I–K) Comparison of the transcript levels (by FPKM values) of proapoptotic genes Cdkn1a (P21), Bbc3 (Puma), and Bax in control (WT) and Cul4bfl/fl-CD4Cre CD4+ T cells. (**P < 0.01, by Student t test). For numerical raw data, please see S7 Data. For supporting data set, please see S2 and S4 Tables. Cul4b, Cullin-4b; TCR, T cell receptor; WT, wild-type. (TIF) [file pbio.3001041.s007.tif]
