# Supplementary material for: The E3 ubiquitin ligase Cul4b promotes CD4+ T cell expansion by aiding the repair of damaged DNA
Source: PLoS Biol. 2021 Feb 1;19(2):e3001041. doi: 10.1371/journal.pbio.3001041 (PMC7888682; doi:10.1371/journal.pbio.3001041)
Supplement: S1 Raw Images — Cells were washed with phosphate-buffered saline (PBS) (Ca2+ and Mg2+-free) and lysed using 1% Triton-X lysis buffer containing 50 mM Tris, 50 mM NaCl, Protease inhibitor cocktail (Roche), Halt phosphatase inhibitor cocktail (Thermo Fisher Scientific), Zn2+-chelator ortho-phenanthroline (o-PA) (LifeSensors), deubiquitylase inhibitor PR-619 (LifeSensors), and 1% NP 40. Protein lysate was harvested and quantitated using Bradford reagent. For Cul4a and Cul4b neddylation experiments, whole-cell lysates were prepared in SDS sample buffer (62.5 mM Tris-HCl, 2% w/v SDS, 10% glycerol, 50 mM DTT, and 0.1% bromophenol blue), vortexed to reduce sample viscosity, denatured by boiling, and then cooled on ice. Samples were resolved on 4%–12% Novex Tris-Glycine Gels (Thermo Fisher Scientific) and then transferred onto PVDF membrane (Amersham Pharmacia Biotech, Piscataway, New Jersey). The membrane was probed with the primary antibodies. Immunostaining was performed using appropriate secondary antibody at a dilution of 1:5,000 and developed with licor odyssey imaging system. (PDF) [file pbio.3001041.s019.pdf]

Figure 1A

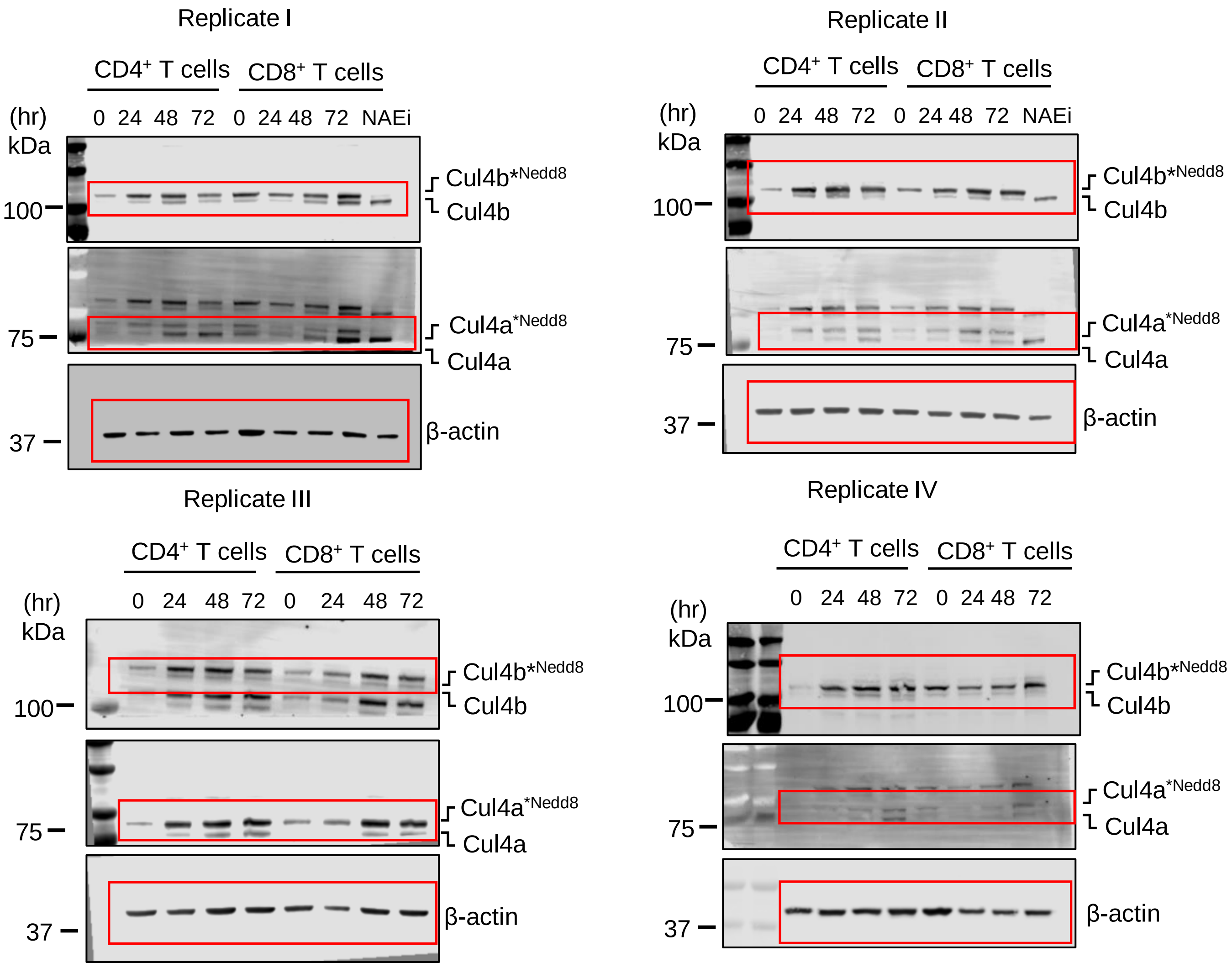

Images were acquired by li-cor odyssey infrared imaging system
Approx. molecular weight ladder (L) was indicated.
PVDF Membranes were cut for immunoblotting of more than one protein. For Cul4a and Cul4b staining blots were stained intermittently, on occasions Cul4a was probed first then Cul4b and vice versa.

Figure 2A

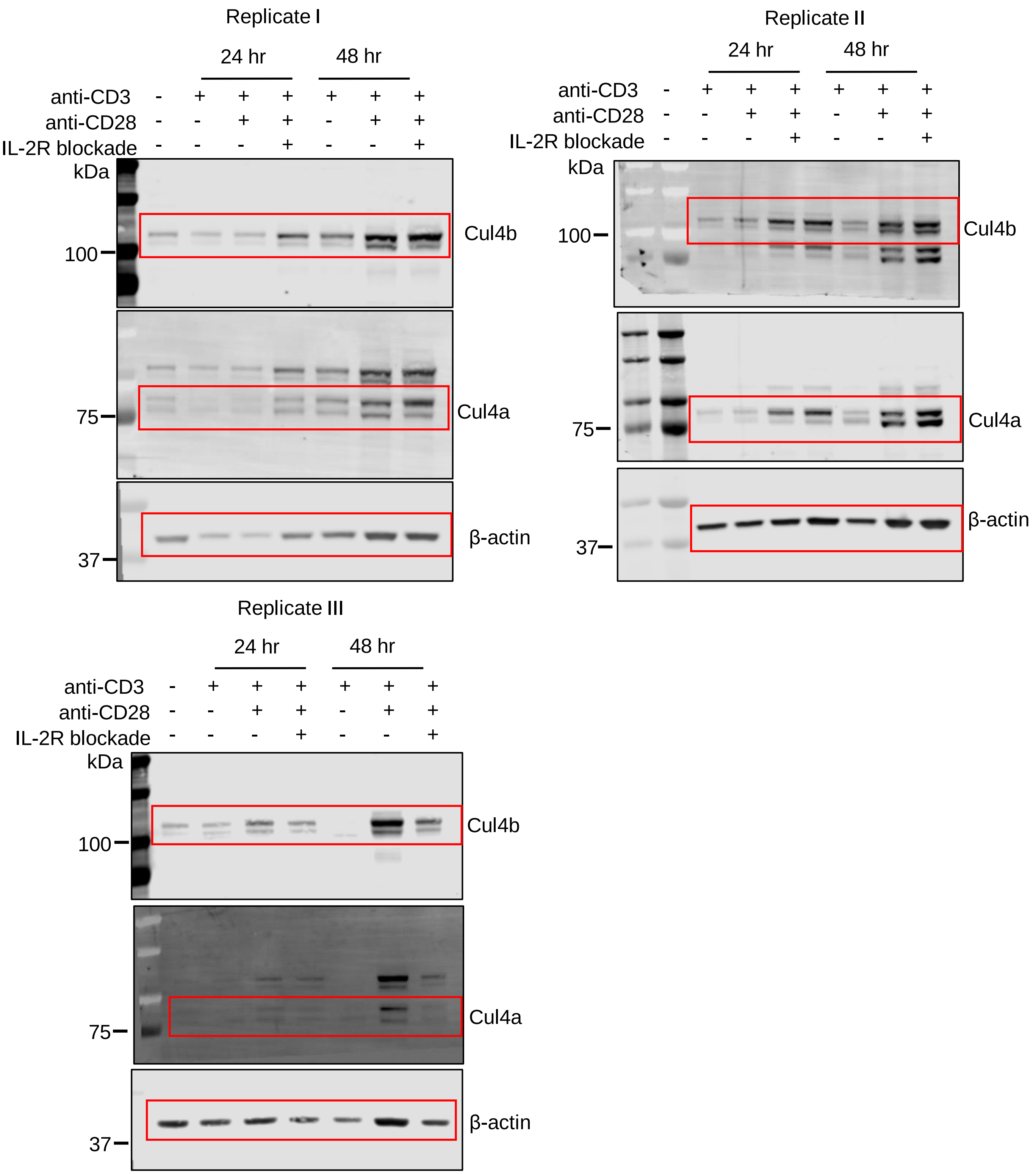

Images were acquired by li-cor odyssey infrared imaging system
Approx. molecular weight ladder (L) was indicated.
PVDF Membranes were cut for immunoblotting of more than one protein. For Cul4a and Cul4b staining blots were stained intermittently, on occasions Cul4a was probed first then Cul4b and vice versa.

Figure 2B

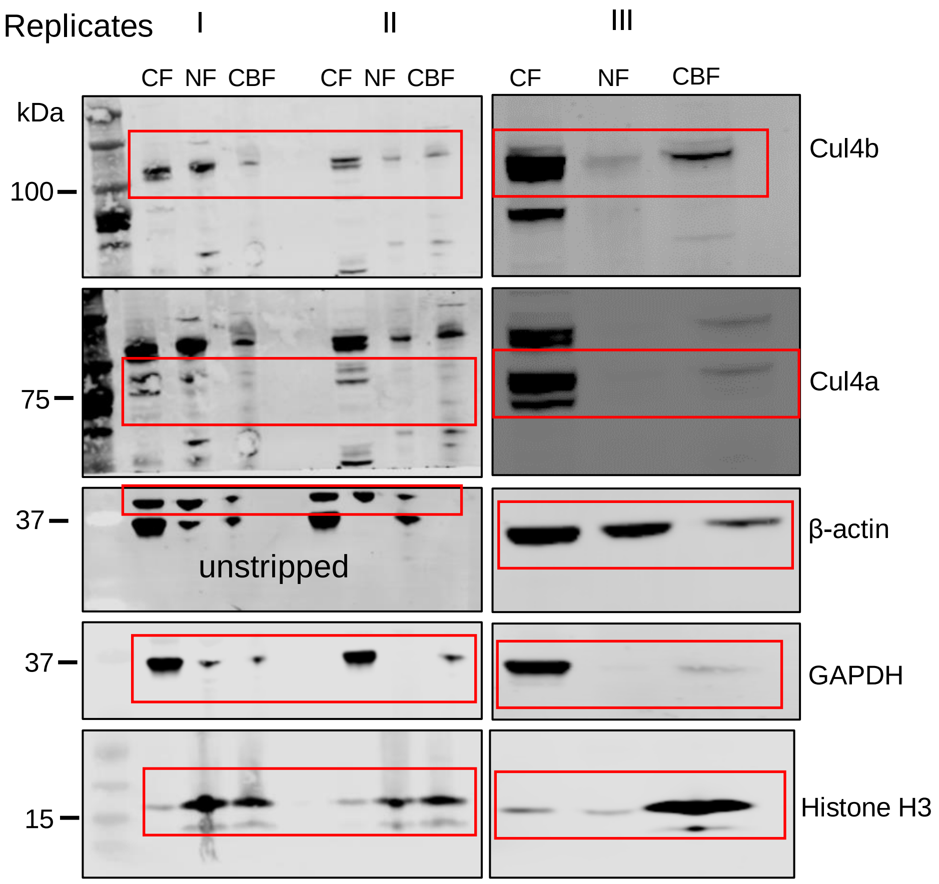

Images were acquired by li-cor odyssey infrared imaging system
Approx. molecular weight ladder (L) was indicated.
PVDF Membranes were cut for immunoblotting of more than one protein. For Cul4a and Cul4b staining blots were stained intermittently, on occasions Cul4a was probed first then Cul4b and vice versa.

Figure 7B

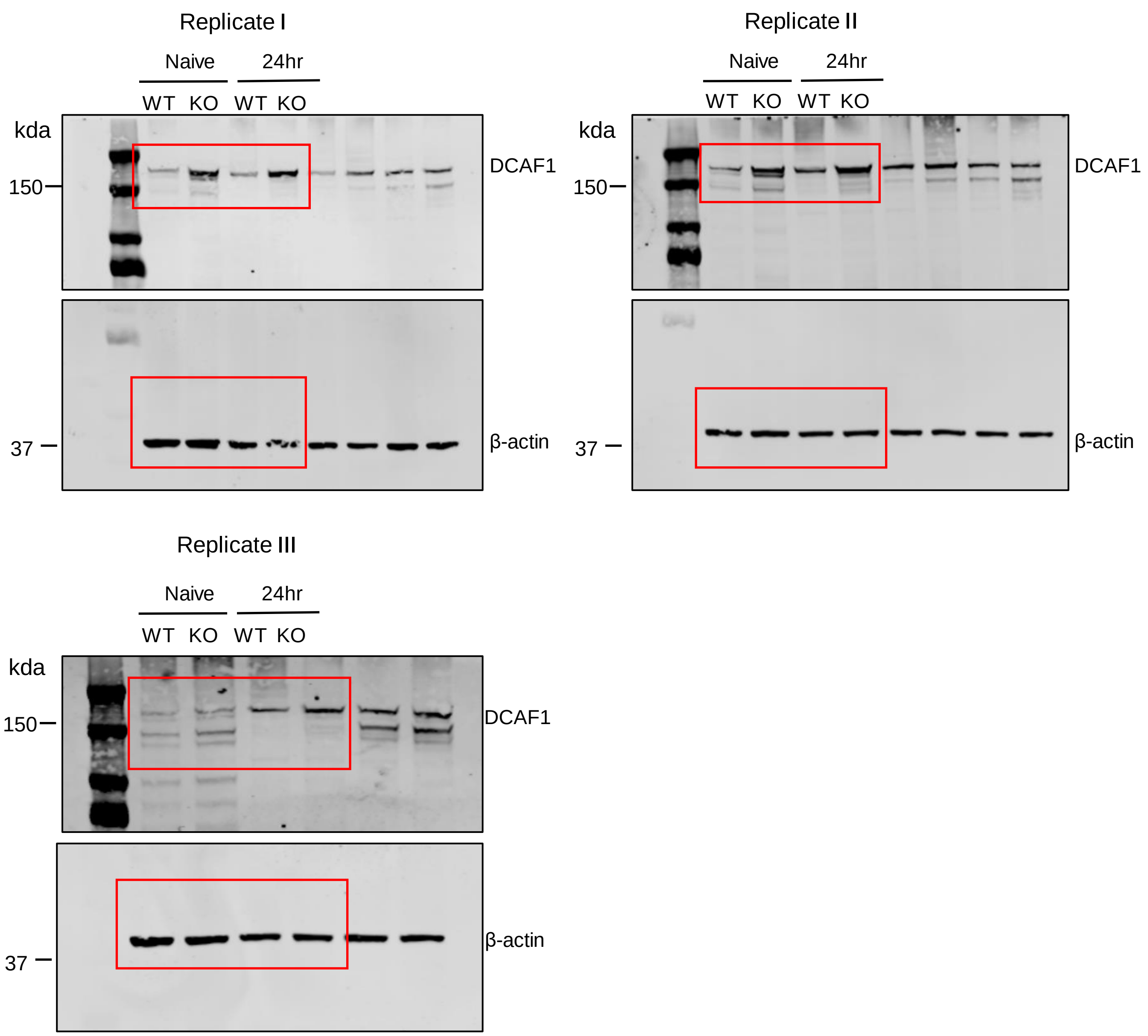

Images were acquired by li-cor odyssey infrared imaging system
Approx. molecular weight ladder (L) was indicated.
PVDF Membranes were cut for immunoblotting of more than one protein.

Figure 7C

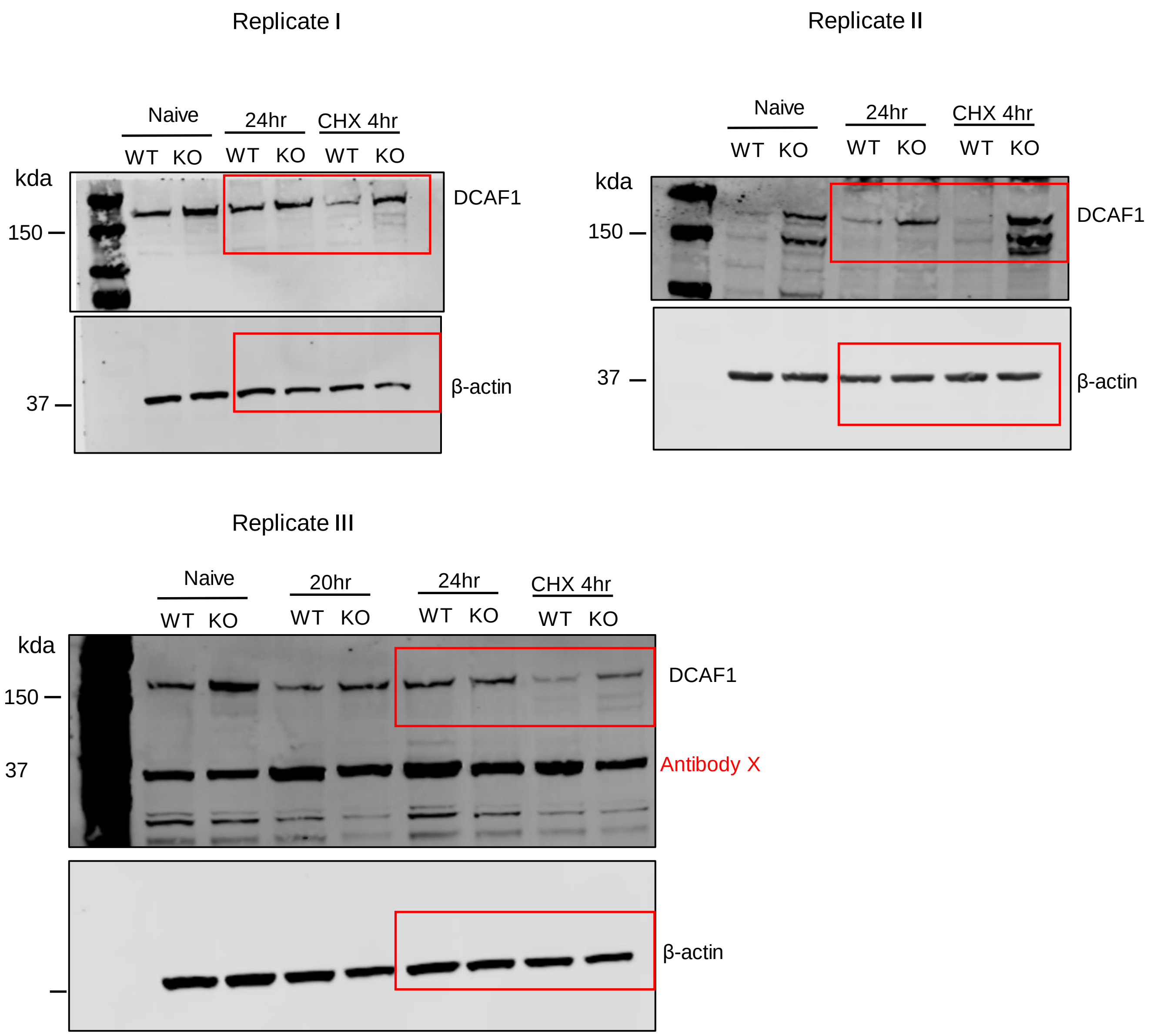

Images were acquired by li-cor odyssey infrared imaging system
Approx. molecular weight ladder (L) was indicated.
PVDF Membranes were cut for immunoblotting of more than one protein.

Figure 7G

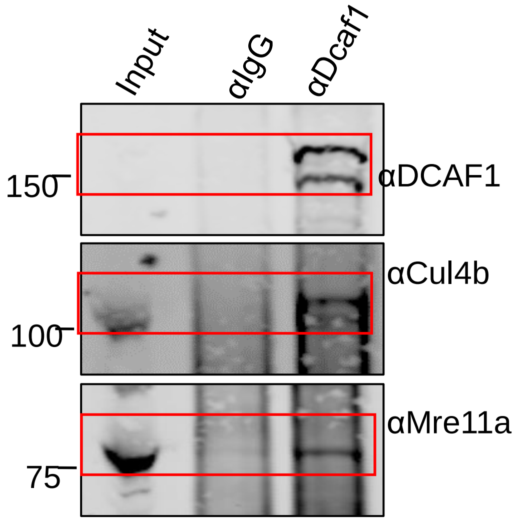

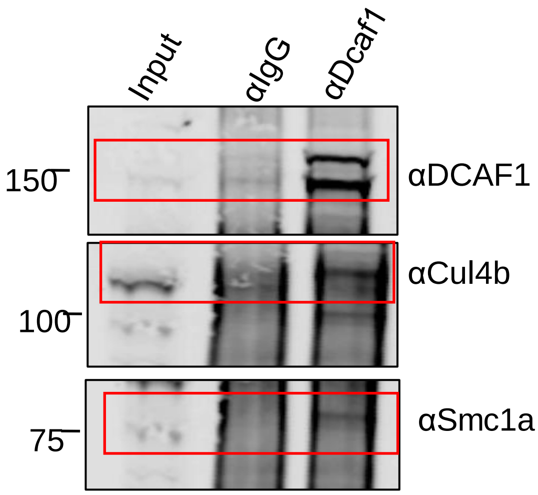

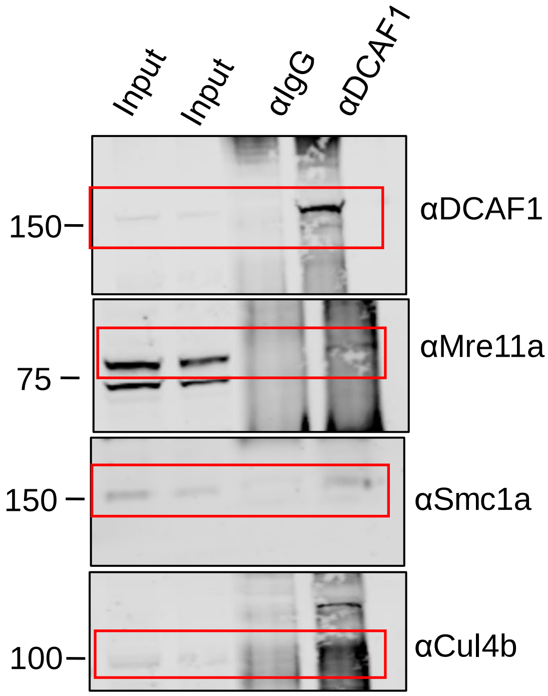

Images were acquired by li-cor odyssey infrared imaging system
Approx. molecular weight ladder (L) was indicated.

Figure 7H

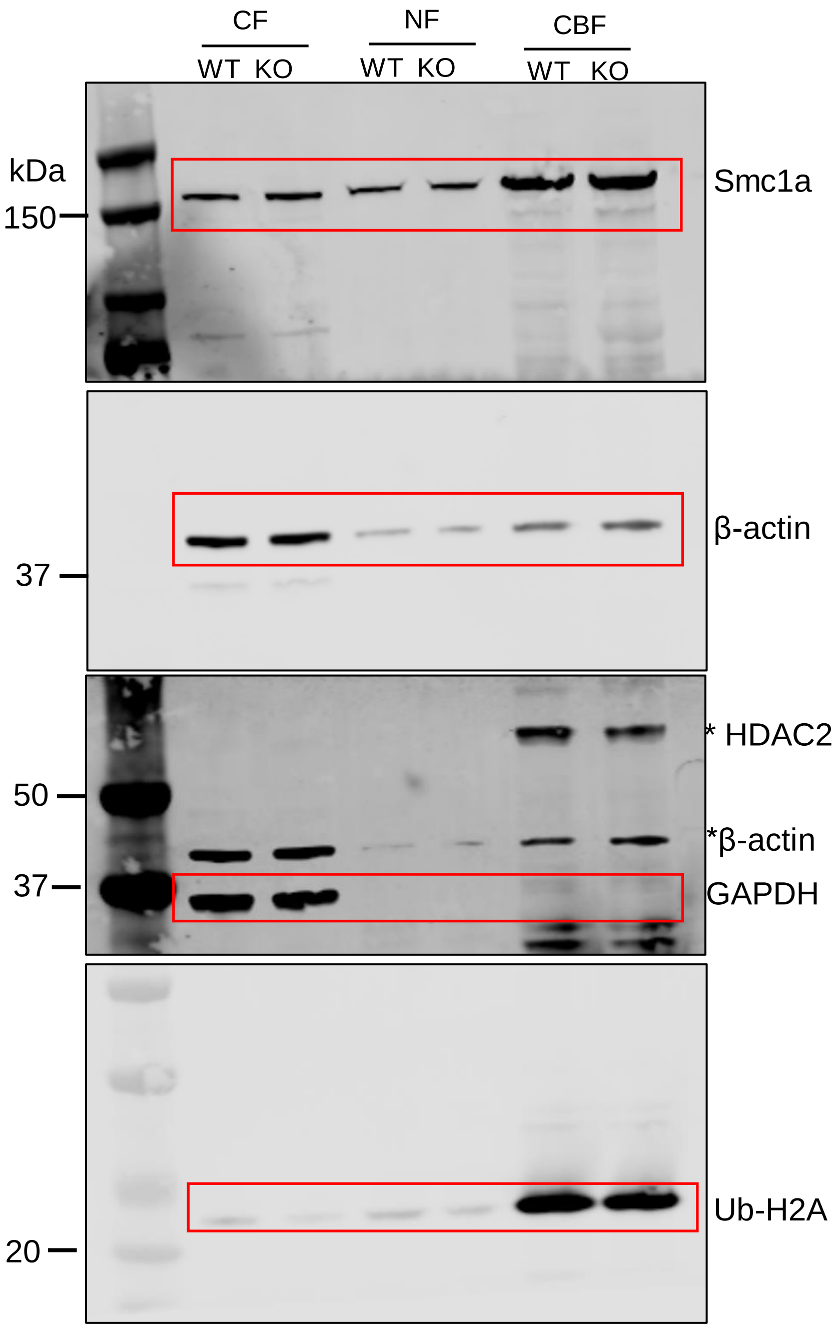

Images were acquired by li-cor odyssey infrared imaging system
Approx. molecular weight ladder (L) was indicated.

Figure 7I

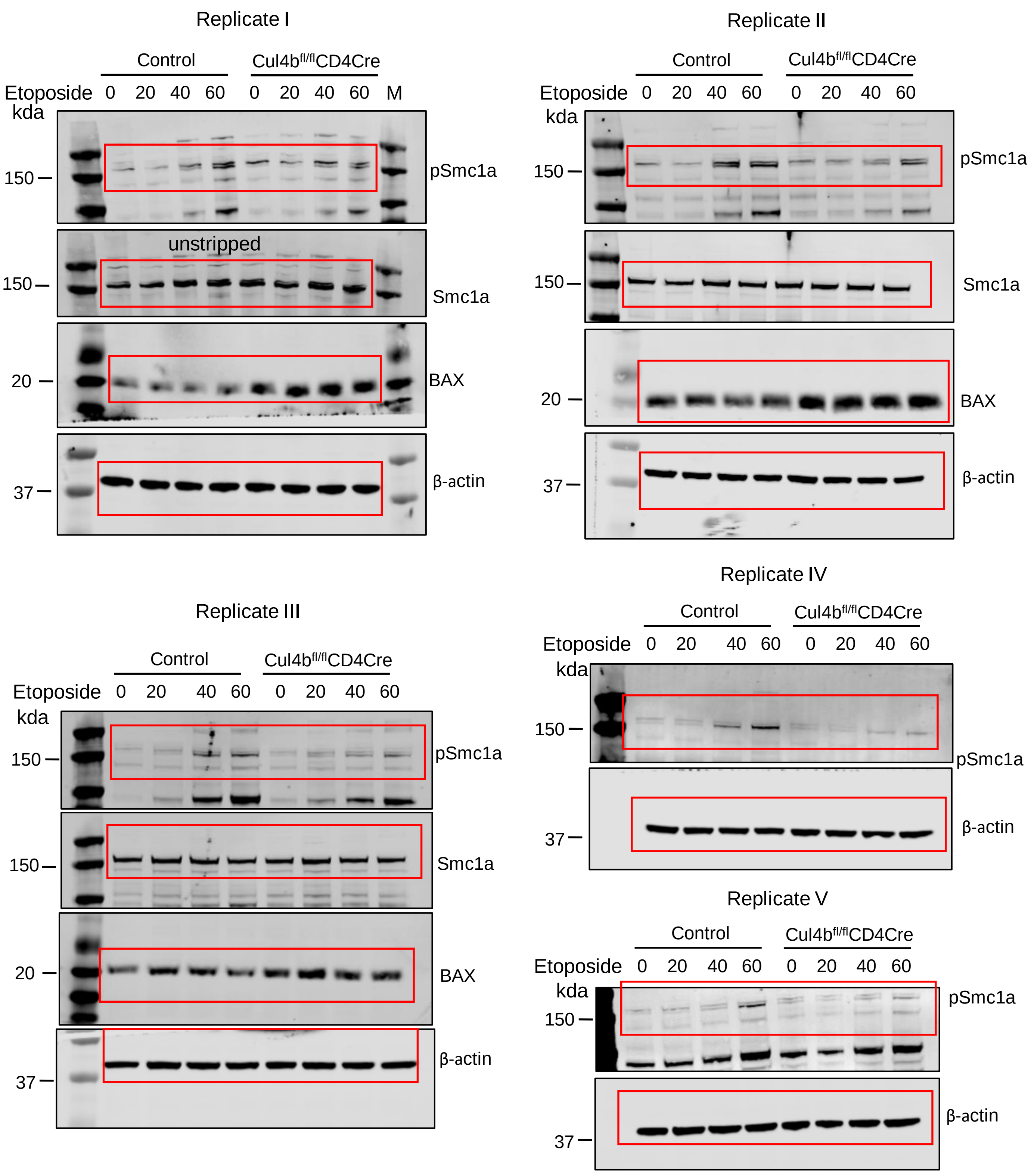

Images were acquired by li-cor odyssey infrared imaging system
Approx. molecular weight ladder (L) was indicated.

Supplementary Figure 1D

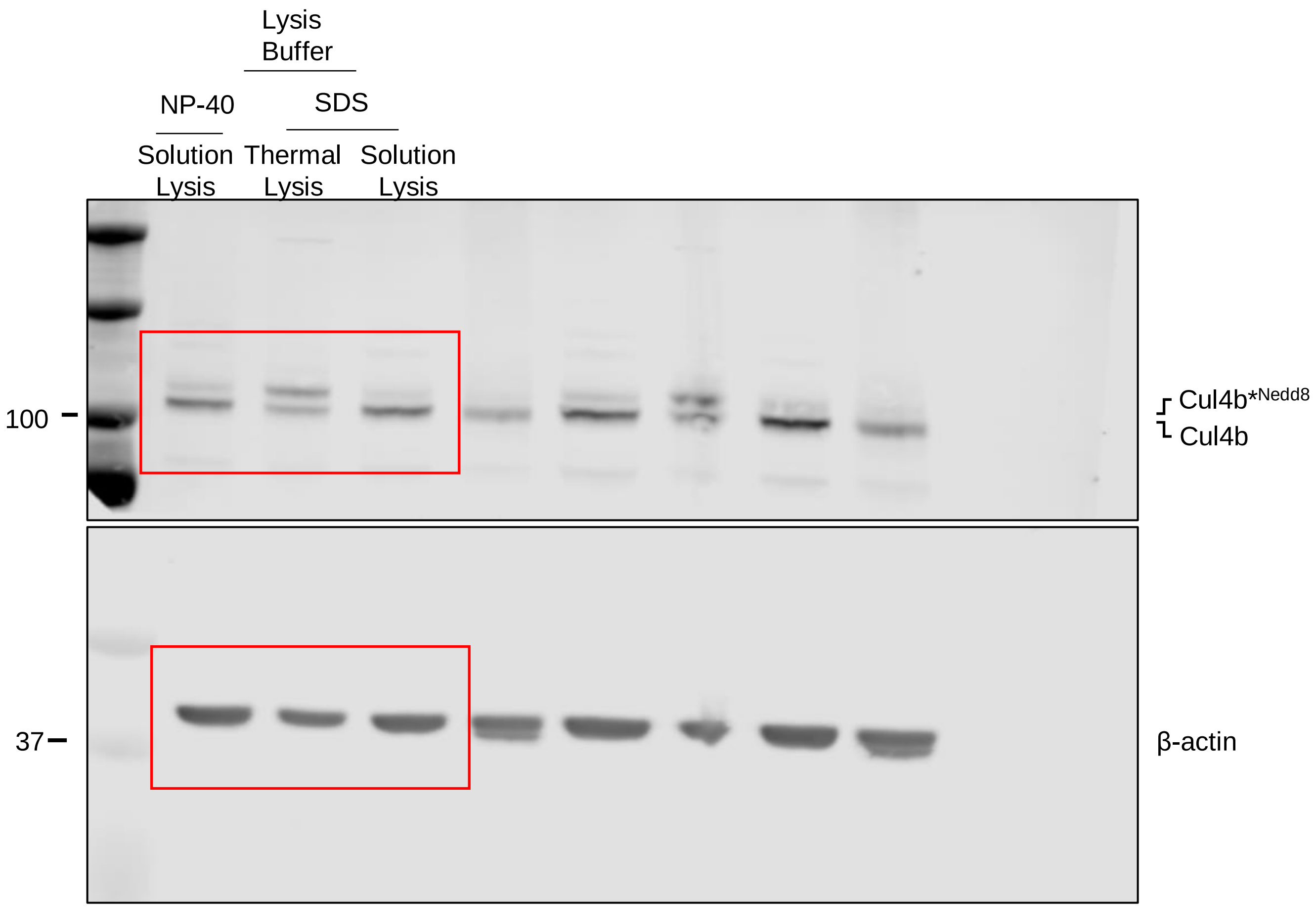

Images were acquired by li-cor odyssey infrared imaging system
Approx. molecular weight ladder (L) was indicated.

Supplementary Figure 7A

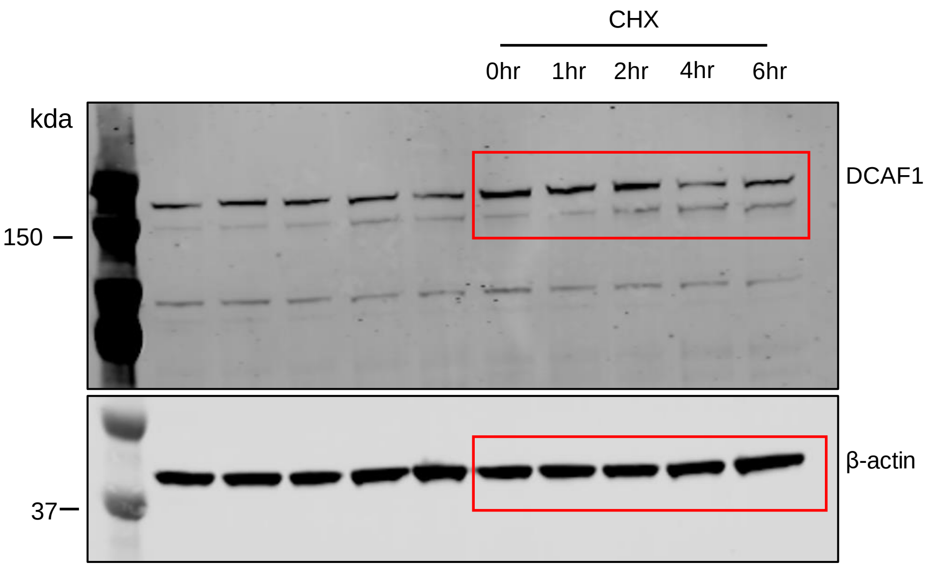

Images were acquired by li-cor odyssey infrared imaging system
Approx. molecular weight ladder (L) was indicated.

Figure 7B

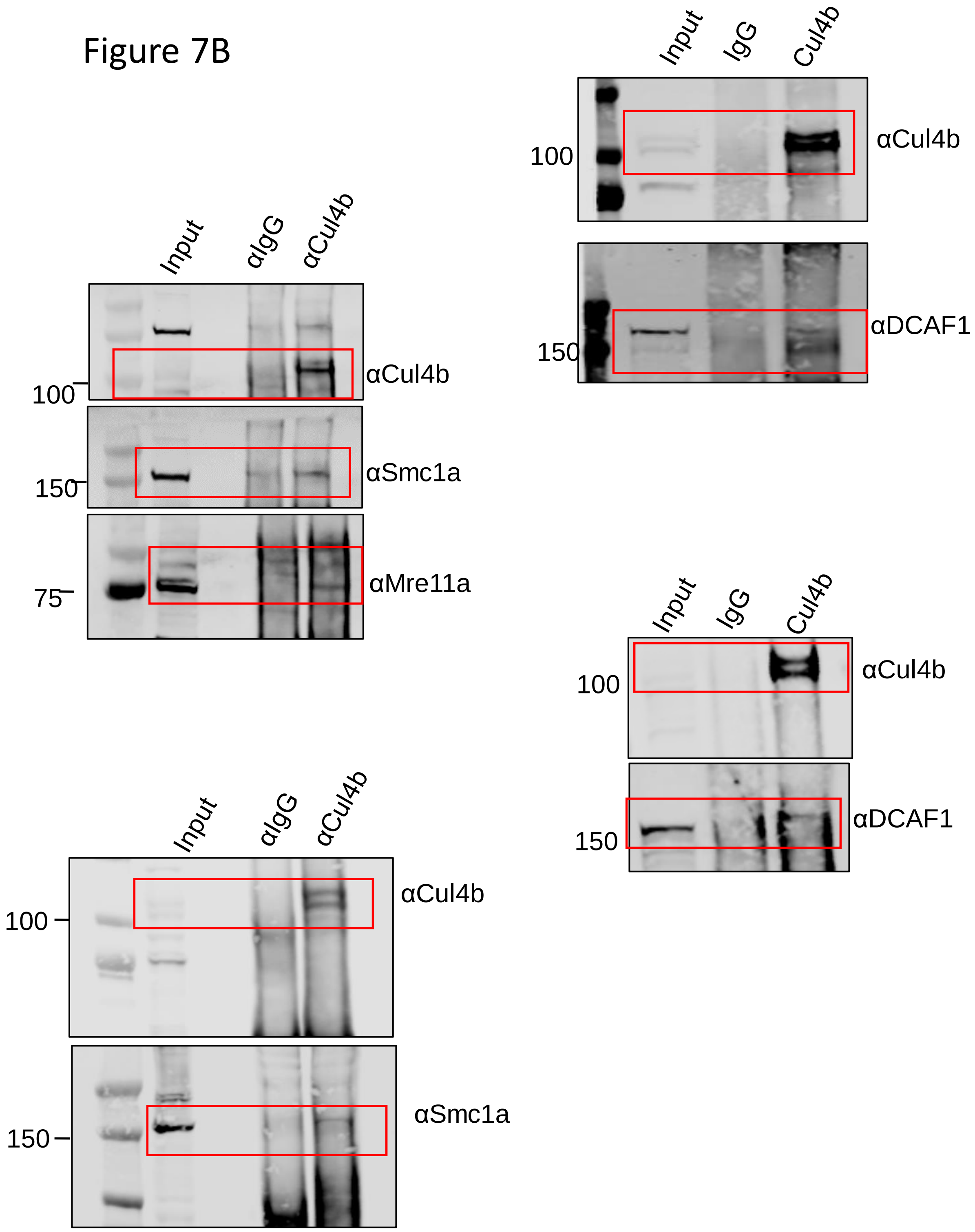

Images were acquired by li-cor odyssey infrared imaging system
Approx. molecular weight ladder (L) was indicated.

Figure 7C

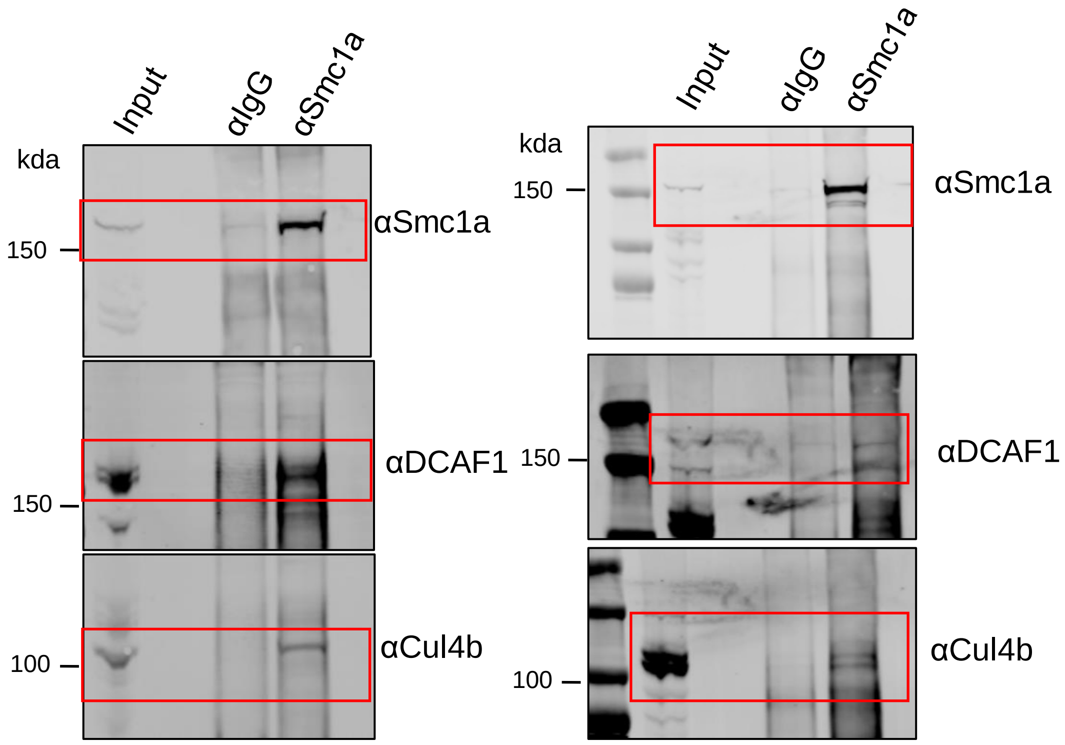

Images were acquired by li-cor odyssey infrared imaging system
Approx. molecular weight ladder (L) was indicated.
