# Supplementary material for: The E3 ubiquitin ligase Cul4b promotes CD4+ T cell expansion by aiding the repair of damaged DNA
Source: PLoS Biol. 2021 Feb 1;19(2):e3001041. doi: 10.1371/journal.pbio.3001041 (PMC7888682; doi:10.1371/journal.pbio.3001041)
Supplement: Attachment — Submitted filename: Reviewers Comments PlosB.pdf [file pbio.3001041.s020.pdf]

REVIEWS:

Reviewer #1: In the present manuscript, Dar et. al. investigate the role of an E3 ligase component, Cul4b, in CD4+/CD8+ T cell activation and expansion during an immune response following T cell receptor (TCR) activation. Transcription/translation of Cul4a and Cul4b were low in naïve T cells, but increased upon T cell activation with anti-CD3/28 antibodies ex vivo, whereas Cul4b seemed much more abundant than Cul4a as estimated by semi-quantitative mass spectrometry. Disruption of the Cul4b gene in murine CD4+ T cells through tissue-specific Cre-mediated LoxP recombination had no obvious effect on naïve CD4+/CD8+ T cells, but affected T cell expansion and activity upon TCR activation in Rag1-/- mice as a model system. Cul4b knock-out cells could still be activated through anti-CD3/28, but failed to expand due to impaired proliferation and higher rates of apoptosis. As the Cul4b knock-out cells also suffer an increase in DNA damage, the authors speculate that Cul4b is involved in the DNA damage response in these cells. Further experimentation identified the DCAF1 substrate receptor as the primary Cul4b-associated factor, suggesting that CRL4DCAF1 is the dominant CLR4 E3 ubiquitin ligase in these cells. Cul4b as well as DCAF1 immunoprecipitation experiments co-purified the Rad50, Mre11a and Smc1a, factors involved in DNA damage response. Depletion of Cul4b does not affect Smc1a recruitment to chromatin, but results in a Smc1a phosphorylation defect, a mark that is implicated in the activation of an S-phase checkpoint.

Overall, the performed experiments together with the obtained results support the majority of the conclusions that are drawn in this manuscript, and the Cul4b knock-out phenotype seems well characterized. As Cul4b is a known DNA damage repair (DDR) component, it is very likely that it carries out similar functions in activated CD4+/CD8+ T cells, although DDR may not be the only pathway that contributes to the observed phenotype. Cul4b is known to interact with more than 20 different DDB1-Cul4-associated factors (DCAFs), which also contribute to functions beyond DDR, e.g. cell cycle progression (i.e. through Cdt2). Disruption of Cdt2 activity is predicted to cause replication re-initiation during S-phase which by itself could lead to the activation of DNA damage checkpoints during S phase, proliferation defects and apoptosis. It is therefore not entirely clear whether increased DNA damage in these cells is (i) caused by the inactivation of CRL4-Cdt2, CRL4-DCAF1 or any other DCAFs, (ii) a consequence of a defective DNA damage response, or (iii) both. Providing the authors balance the interpretation and discussion of their findings in the light of these uncertainties, this reviewer would support publication in PLOS Biology.

Specific comments:

Is it possible that the contribution of Cul4b to T cell proliferation might also be governed by other DDB1-Cul4-assocated factors (DCAFs) such as Cdt2, a CRL4 substrate receptor that controls entry into S phase through degradation of the replication licensing

factor Cdt1? Could DNA damage be a result of the Cul4b-/- cells failing to inactivate Cdt1 in S phase leading to replication re-initiation?

We thank reviewer for the comment. We acknowledge the importance of other substrate receptors such as CDT2 in DNA damage. While analyzing for the possible substrate receptors (SRs) which could explain the phenotype, we took multiple things into consideration, for example concentration of substrate receptors, fold change in the concentration of the SRs between control and Cul4b^{cKO} cells, and extent to which each substrate receptor was immunoprecipitated with Cul4b (based on peptide count). We observed that the concentration of DCAF1(88.5nM) in T cells far exceeded that of CDT2 (3.3nM) (Supplementary Figure 6A). The amount of DCAF1 immunoprecipitated with Cul4b was also more than for CDT2 (12 versus 1 peptide for CDT2). Thus, we posit that DCAF1 might be major but not the sole contributor of the phenotype in T cells. In keeping with this reviewer's point, we did find an ~3-fold accumulation of CDT1 in Cul4b^{cKO} cells (Log2 fold change of 1.7, Supplementary file 1) which can induce DNA Damage if not degraded during cell cycle progression. We have added comments to the discussion (line 512-518 highlighted in yellow) detailing that Cul4b has potential to regulate DNA damage response through multiple pathways.

Western blots: How reproducible are the findings? To this reviewer it seems that claims of Figure 1a do not reproduce in Figure 2a. The relative abundance of unmodified versus neddylated Cul4b and Cul4a is not comparable between these two figures.

As shown in the graphs for Figure 1, this is highly reproducible in the four replicates performed for this Figure panel (Western blots of each replicate are included in the additional data file). However, the data shown in Figure 2a showed a bit more variability in part due to the death of cells stimulated with anti-CD3 alone (specifically at 48 hours). In the replicates for Figure 2a we also found some variability with the ratio of neddylated and un-neddylated Cul4. It should be noted that neddylation and de-neddylation are highly sensitive processes regulated in part by the COP9 signalosome. Inhibition of this complex is important to accurately analyze the neddylation of Cul4. Both buffer composition and incubation time greatly influences the extent of inhibition of the COP9 signalosome and thus neddylation of Cul4. This required significant optimization to allow an accurate analysis of the neddylated and de-neddylated versions of Cul4a and Cul4b (Supplementary Figure 1D). The replicates performed for Fig1a were done subsequent to this analysis, while those for Figure 2a were not. However, we point out that the experiments and our conclusions for Figure 2a are focused on the stimuli that drive expression of Cul4a and Cul4b, this should not be impacted by the buffer conditions. We have added emphasis to the importance of buffers and conditions to the discussion of lysis conditions in the materials and methods section (line 830-834 highlighted in yellow).

In Figure 7B, DCAF1 levels markedly differ between WT and Cul4b KO cells after 24h stimulation, whereas in Figure 7C this difference is only detectable upon cycloheximide treatment, but not in the control. How would the authors explain this inconsistency?

We would like to point out that in figure 7C the loading control (β-actin) in lane 1 and 2 is also different. To assess the changes in DCAF1 levels in Cul4b-deficient cells, we quantified the levels of DCAF1 in multiple replicates and normalized the data to β-actin. As seen in Figure 7D, we observed the difference between WT and Cul4b KO cells. The raw data for the replicates of these and other experiments are included as an additional data file.

Also, does proteasome inhibition through MG132 restore DCAF1 levels in WT cells?

While we have strong evidence that DCAF1 is regulated by ubiquitin mediated degradation, we do not show whether this is due to proteasomal or lysosomal degradation. Given that Cul4b is known to drive either K48 ubiquitination or monoubiquitination, we predict that DCAF1 is degraded/cleaved by the proteasome. Given that MG132 can prevent TCR signaling, and that TCR signaling is needed to promote the expression and neddylation of Cul4b, interpreting experiments with MG132 will be difficult to interpret. Thus, we have added text to this effect while not ruling out that other means of degradation might be at play (line 333-335 highlighted in yellow).

Figure 7G: IP westerns are not too convincing. For example, how reproducible is the co-immunoprecipitation of Smc1a? Smc1a seems hard to detect, but appears to be almost equally abundant in Suppl. Fig. 6E in the IgG control. Why are there two bands for DCAF1 in Fig. 7G, but only one in Figure 7B and C and Suppl. Fig. 6E? Can the authors be sure that the bands detected by their antibodies in Fig. 7G and Suppl. Fig. 6E indeed correspond to the appropriate target protein?

Comments regarding DCAF1 - In these immunoprecipitations, to be as biologically relevant as possible, we used primary cells. To generate sufficient protein for this analysis, we stimulated primary CD4 T cells for 48hrs to get 2-3 mg protein per condition. While these interactions appear weak, we have repeated these experiments multiple times and observed these interactions by two complementary but different methods. In one method, we used IP MS/MS. When we IP’ed Cul4b, we identified many peptides for DCAF1 (no peptides were identified in the IgG control IP). Additionally, when we IP’ed DCAF1, we ID’ed Cul4b via MS/MS. We then analyzed a Cul4b IP via immunoblot we were able to validate the interactions between Cul4b and DCAF1.

The reviewer is correct that in Figure 7B there is one prominent band for DCAF1 while in 7D, there are two bands. The only difference that may account for this is that 7B was done at 24 hours after TCR stimulation while 7D is performed after 48 hours. The multiple bands of DCAF1 likely correspond either to different splice variants of DCAF1 or cleaved product. We have evidence that it might be the cleaved product and importantly, while we have observed this phenomenon consistently, the biological significance of these products remains unknown. This would be an exciting thing to study but it is not the focus of this manuscript. We have made comments about this in the results section (Line 356-358 highlighted in yellow).

Comments regarding Smc1a. Again, while these interactions appear weak, we have repeated these experiments multiple times and observed these interactions repeatedly and by two complementary but different methods. The reverse Co-IP was preformed which also confirmed the interaction of Cul4b and DCAF1 with Smc1A (data is included as Supplementary Figure 7B). We have included text regarding this in the result section (Line 355-357 highlighted in yellow). Further, in the case of the interactions with Smc1a, we found Smc1a associated with both DCAF1 (5 peptides) and Cul4b (6 peptides), but did not identify Smc1a peptides in the IgG isotype control. It is worthwhile noting that these interactions are all the more relevant considering that Smc1a was unlikely to be identified due to non-specific interactions since it was identified in both the IP's, using different antibodies, but not in the control. This was then validated using IP western blot. We have now replaced the SMC1A with the better blot (in Supplementary figure 7A). While we could likely achieve more dramatic results using cell lines with overexpressed proteins, the data we provide, using these two methods, are more likely to be biologically relevant. All the IPs performed are included in the additional data file.

Minor comments:

p. 5, third paragraph: Figure SB probably refers to Figure S2B

We thank reviewer for this comment, the figure label has been corrected from SB to S2B

p. 9, second paragraph: the authors state: '...given the abundance of pATM and g-H2AX staining in Cul4b-deficient T cells, it was clear that the MRN complex was not defective (Figure 7D-G). ...', but no data is not provided.

The data we were referring was from figure 6D-G rather than 7D-G. we have done the changes in the text and is highlighted in yellow

Supplementary Fig. 6A: I think the authors mean -Log10(p value) in the second column, not +Log10(p value).

The necessary changes have been done in the table

Reviewer #2: Manuscript ID: PBIOLOGY-D-20-00560R1
Title: Cul4b promotes CD4 T cell expansion by aiding the repair of damaged DNA
Authors: Dar et al.

The manuscript by Dar et al. builds on previous work by this laboratory discovering the upregulation of Cul4b in T cells following TCR activation to further describe the expression pattern of Cul4a and 4b as a function of T cell stimulation, the effect of conditional loss of Cul4b in CD4 T cells, the relative abundance of DCAF substrate receptors in the absence of Cul4b, and identification of potential interaction partners of Cul4b in T cells.

Overall, this is a solid body of work whose conclusions are, for the most part, supported by the experimental results. The finding that loss of Cul4b in T cells impairs T cell proliferation and survival without marked loss of cellularity, and is somewhat milder in phenotype than mice with conditional loss of DCAF1 in the T lineage is interesting, although the general phenotypes including defects in DNA repair, cell cycle progression, and increased apoptosis reported here have been observed in non-immune cell types, so they are not particularly surprising or novel. The identification of DCAF1 as a major Cul4b substrate interacting protein in CD4 T cells and the stabilization of DCAF1 upon loss of Cul4b provides strong evidence that DCAF1 serves as a major substrate receptor for Cul4b, but this work doesn't shed much light on what the cellular targets of the Cul4b-DDB1-DCAF1 E3 ligase might be. The attribution of greater DNA damage in Cul4b-deficient CD4 T cells as being due to its association with DDR factors is felt to be an overinterpretation, as the evidence for these interactions is not very convincing. Furthermore, the suggestion that Cul4b "preferentially associated with DCAF1" (pg. 4) may be somewhat misleading, as this conclusion is based mainly on pull-down assays that can be biased for abundant DCAFs rather than any systematic analysis of affinity for the large number of DCAFs identified as interacting with Cul4b. There were also some puzzling features of some of the experimental data that need fuller explanation. These and other concerns are described further below.

Major concerns:

1. It would be important for the authors to evaluate the T cell repertoire in the Cul4b conditional knockout mice, given evidence that loss of DCAF1 in B cells or T cells alters the immune repertoire (e.g. in B cells by dysregulating RAG1 levels and in T cells possibly interfering with DNA repair). This may or may not be masked by redundancy with Cul4a. This would also have implications for understanding the outcome of the adoptive transfer experiments in RAG1-/- mice.

We thank the reviewer for this comment regarding the published role of DCAF1 in dysregulating RAG1 levels and DNA repair. We would like to mention that we have used CD4-Cre to delete Cul4b in T cells at the double positive (DP) stage. DP cells that become mature CD4 SP and CD8 SP cells will lack Cul4b. The rearrangement of TCR genes takes place before T cells enter the DP stage prior to Cul4b deletion. So, while exploring the role of Cul4b in T cell repertoires would be interesting, these conditional knockout mice would not be an appropriate model for such studies. Supporting this, our data shows there is little variability in the percentages of CD4 T cells and also no difference in the expression of CD3.

2. There is a concern that undue emphasis was given to characterizing DCAF1, when levels of other DCAFs seem to be more strongly affected by loss of Cul4b. Furthermore, there is no biochemical evidence that DCAF1 "preferentially associates" with Cul4a, which could reflect a bias introduced by relative DCAF abundance.

We thank reviewer for the comment. We do acknowledge that other substrate receptors might be important in regulating the T cell function and proliferation. To narrow down for the possible substrate receptor which could explain the phenotype, as mentioned above, we took multiple things into consideration like concentration of substrate receptors, fold change difference in the concentration of these receptors between control and Cul4b cKO cells and number of peptides co-immunoprecipitated with Cul4b. We identified 4 substrate receptors that were increased > 2 fold in Cul4b deficient T cells, DCAF1 was among these (Supplementary Figure 6A). Further, the number of DCAF1 peptides immunoprecipitated with Cul4b were higher compared to other DCAFs (Figure 7E). With all these observations into consideration, we posit that DCAF1 might be a major but not the sole substrate receptor in proliferating T cells. Further addressing the issue of the bias due to relative abundance, it is interesting to note that despite the higher concertation substrate receptors like DCAF7, DCAF8, DCAF13 and DDB2, these factors were less immunoprecipitated than DCAF1. The number of peptides IP'ed for DCAF1, DCAF3, DDB2, DCAF8, DCAF7 were 12, 10 ,7, 2 and 1 peptides respectively.

One more interesting note regarding DCAF1 was that it was also less ubiquitinated in Cul4b KO T cells compared to WT cells (data included in revised figure 7H). Thus, we believe it is a top candidate to be studied in the context of T cell proliferation. Nevertheless, we have dropped the word 'preferentially' from the text.

3. A major weakness of the study is the relative lack of characterization of the transcript levels for the proteins identified as being up- or down-regulated by loss of Cul4b. The implicit assumption is that Cul4b is regulating protein turnover, but the effect could be indirect through transcriptional regulation.

We appreciate the reviewer for raising the point. We would like to add that most of the proteins which were either up or down regulated were unchanged at mRNA level. We agree with the reviewer that effect of Cul4b deletion could have indirect effects and alter transcriptional regulation. supporting this, we did find the enrichment of genes associated with pathways like cellular response to DNA damage stimulus, Cell cycle processes and intrinsic apoptotic signaling response in cells lacking Cul4b. To address this concern, we have now included the fold change differences in transcript levels for the most relevant proteins. In Supplementary Figure 7B the transcript levels of substrate receptors are shown. Further in supplementary Figure 7C-G, transcript levels of proteins associated with the DNA damage response are shown and in supplementary Figure 7I-K transcript levels of pro-apoptotic proteins are shown. Requisite changes in the results, material and methods, and figure legends sections are highlighted in yellow at lines 320-323, 359-362, 389-391, 1021-1022, 1106-1102, 1106-1110, 1120-1122.

4. The IP data suggesting Cul4b specifically associates with SMC1a and MRE11A is unconvincing (specificity/robustness in isotype control vs specific IP), poorly described, and needs further biochemical validation, including reciprocal co-IPs. In the absence of

this, a direct link between Cul4b and DDR is deemed very tenuous, which may require revising this major conclusion and the title.

Comments regarding SMC1A and MRE11A: In these immunoprecipitations, to be as biologically relevant as possible, we used primary murine T cells. To generate sufficient protein for this analysis, we stimulated primary CD4 T cells for 48hrs to get 2-3 mg protein per condition. While these interactions appear weak and transient, we have repeated these experiments multiple times and observed these interactions by two complementary but different methods. In one method, we used IP MS/MS. When we IP'ed Cul4b, we identified many peptides for MRE11A and SMC1A (no peptides were identified in the IgG control IP. Importantly, we found Smc1a associated with both DCAF1 (5 peptides) and Cul4b (6 peptides), but did not identify Smc1a peptides in the IgG isotype control. It is noteworthy that Smc1a was identified in both the Cul4b and DCAF1 IP's, that used different antibodies, but not in the control. This was then validated using Cul4b IP and western blot. The reverse co-IP was preformed which also confirmed the interaction of Cul4b and DCAF1 with Smc1A (data is included as Supplementary Figure 7B). We have included text regarding this in the results section (Lines 355-356 highlighted in yellow). We also replaced blot for SMC1A (in Supplementary figure 7A). All the data showing the interactions of Cul4b, DCAF1 and SMC1A are included in the additional data file. While we could achieve more dramatic results using cell lines with overexpressed proteins, the data we provide, using these two methods, are more likely to reflect a biologically relevant interaction between these two proteins.

The other confounding issues is loss of neddylation in case of Cul4b in the lysis buffer used for IP.

5. Figure 4b. The Cul4bfl/fl CD4Cre mice seem to show an accumulation of CD4lo CD3- T cells which is not discussed. Is it possible that Cul4b regulates CD3 expression?

To the best of our knowledge and observation we did not find any difference in the CD3 expression, this might be the gating artifact and we have replaced the graph with one that is more representative.

6. There was a general absence of specific description of antibody reagents used for IP, western, and flow cytometry experiments (source, clone, etc). These should be included for transparency.

We thank reviewer for raising the issue, we have updated the manuscript and have included all the information about the reagents used in the study. Changes are highlighted in yellow in the material and method section

Minor concerns:

1. Figure 1. Both the neddylated and non-neddylated forms of Cul4a and Cul4b

appear to increase in response to T cell activation. The graphs are plotted for each form, but this reviewer thinks it would be instructive to examine the ratio of the two forms.

As suggested by the reviewer we have included the ratios of the two forms. The data is included in the figure 1E-F

2. Figure 5d. The presence of a fairly large number of CD45.1-CD45.2 double-positive cells in the lymph nodes is puzzling. Is this a gating artifact or non-specific staining?

We revisited our flow cytometry files and found that it a gating artifact and replaced the flow plot.

3. Figure 6 D-J. The flow data should include specificity controls for intracellular staining, including isotype and fix-no perm controls for rigor and transparency.

For flow cytometry staining, FMOs, isotype controls, and internal staining controls were all used. For the simplicity and avoiding crowded figures only unstimulated and stimulated cells were shown in the figures. We have updated the figures with the fluorescence minus one (FMO) controls in figure 6D-J. The specificity in staining can also be inferred from the plots of unstimulated cells in which staining is low but increases upon TCR stimulation. The etoposide treatment further elevates the expression of the proteins analyzed in figure 6D-J. The necessary changes are highlighted in yellow for lines 659, 729-730.

4. The adoptive transfer of T cells to establish colitis in RAG1-/- mice is not particularly well justified, experimentally controlled, or interpreted. Why did the authors choose this approach rather than testing model T cell antigen-dependent immune responses? The experiment did not include a vehicle injected control, but only measures response against a positive control (Cul4bfl/fl T cells). Did the RAG1-/- animals receiving Cul4bfl/fl CD4Cre T cells show any evidence of inflammation compared to a negative control (i.e. vehicle injection)? The assertion of the authors that "Cul4b was required for the expansion and pathogenicity of activated CD4 T cells" (pg. 6) is an over-interpretation in the absence of negative control, and further data regarding immune repertoire diversity explained in Major Concern #1.

The T cell transfer model of colitis is a useful tool to assess the function of activated T cells in vivo and is a model for chronic intestinal inflammation. It involves adoptive transfer of naïve CD4 T cells ($CD4^+CD25^-CD44^-$) into immune-deficient Rag KO mice. There are numerous published reports using this method to test how genetically altered CD4 T cells drive inflammation (Claesson, Bregenholt et al. 1999, Leppkes, Becker et al. 2009, Ostanin, Bao et al. 2009, Mohammad, Starskaia et al. 2018). Rag1-/- mice lack T cells, hence there will be no T cells in these mice prior to T cell transfer. Given that there would be no cells to analyze in a vehicle control, we compared the T cells in recipients receiving WT or Cul4b-deficient T cells. We have added a justification for the T cell mediated colitis

in the text. We further included data on Ki67 to confirm that loss of Cul4b impacted T cell proliferation in vivo. The frequency of Ki67 among Cul4bfl/flCD4Cre CD4 T cells was lower than that of control CD4 T cells in both spleen and colon indicating that Cul4b is required for an active expansion of CD4 T cells in secondary lymphoid compartments. The necessary changes in text are highlighted in yellow on lines 202-204, 216-221.

5. What is the source of the doublet in the DCAF1 western blots? The detection of one or two bands seems to vary under different conditions/in different experiments (e.g. Fig. 7C vs 7G).

The reviewer is correct that in Figure 7b there is one prominent band for DCAF1 while in 7D, there are two bands. The only difference that may account for this is that 7D was done at 24 hours after TCR stimulation while 7D is performed after 48 hours. The multiple bands of DCAF1 could likely correspond to different splice variants of DCAF1 or cleaved product. We have some evidence that it is a cleaved product. Importantly, while we have observed this phenomenon consistently, the biological significance of these two forms remains unknown. This would be an exciting thing to study but it is not the focus of this manuscript. We have made comments about this in the results section (Line 356-358 highlighted in yellow).

6. Figure 7I purporting to show poor SMC1A activation (pSMC1A) in response to stimulation cannot be properly interpreted without a total SMC1A blot as a control.

We thank reviewer for the comment, we did repeat the experiment and stained the blots with Total SMC1A. The Total SMC1A is included in the revised figure 7J. The raw data of all the replicates for the experiment are shown in the additional data file.

Reviewer #3: In this paper, the authors address a long-standing question on how T cell activation regulates the rapid cell proliferation. Following their previous proteomic studies that identified Cul4b being actively modified by neddylation, an indicative of functional activation, they focused on the role of Cul4b and its paralog Cul4a and their major substrate receptor, DCAF1, during T cell activations. They showed in this paper that during T cell activation, the steady state level and neddylated form of Cul4b was increased. Using a conditional Cul4b strain, they found that deletion of Cul4b impaired T cell proliferation and survival, and accumulated DNA damages. They further identified that Cul4b and its major binding partner, DCAF1, interact with multiple proteins involved in sensing and repair of DNA damage. SMC1A, a structural maintenance of chromosomes protein and a component of cohesin that is linked to the ATR/ATM DNA repair pathway, was implicated as a potential
downstream factor of Cul4b.

Overall, this is an important topic and most data they presented are clean. However, the

paper, at its present form, is mere collection of some phenotypic descriptions and lack any mechanistic novelty. Multiple studies have demonstrated that the function of Cul4b, and DCAF1, is critical important for cell proliferation, survival and animal development. The role of Cul4b in DNA damage has been extensively characterized. The role of DCAF1 in T cell activation was also carefully characterized. Identifying Cul4b- and DCAF1-interacting proteins, as shown in Figure 6 and 7, is a good start, but the current study falls far short to provide novel insight without identifying the key or relevant protein(s) or substrate(s) that can explain the function of Cul4b in T cell activation. For example, is SMC1A a substrate of Cul4b or DCAF1? And is the regulation of DNA repair the main function of Cul4b during T cell expansion? Cul4b-deficient T cells apparently suffer significant defects during activation even in the absence of etoposide.

1. Fig.5 claimed that "Cul4b promotes the maintenance of CD4 effector (CD44hiCD62Llo) T cell numbers" from the BM adoptive transfer model. However, this observation may be caused by a higher proliferation rate of control activated T cells when compared to Cul4b deficient cells.

We thank reviewer for the comment. We agree with the reviewer that higher proliferation rate of activated T cells might be responsible for higher numbers. However, we can’t ignore the observation that control T cells are also surviving better. That is why we think Cul4b is required for homeostasis and maintenance of activated CD4 T cells.

2. Fig. 7 concluded Cul4b-DCAF1 complex in activated T cells may be involved in protein degradation as can be seen by higher DCAF1 level in Cul4b deficient cells but later they demonstrated SMC1A was phosphorylated rather than degraded by Cul4b-DCAF1 complex. Is the SMCA1 phosphorylation related to Cul4b or DCAF1 in any way? How does Cul4b deletion impairs SMC1A phosphorylation after exposure to etoposide?

Reviewer has raised an excellent question that how Cul4b or DCAF1 regulate SMCA1A phosphorylation. To address this, we did carry out the diglycine remnant profiling to find out what are the proteins that are ubiquitinated by Cul4b in CD4 T cells. Then we used this dataset and analyzed the overlap between the protein interacting with Cul4b and DCAF1 with differentially ubiquitinated proteins. Interestingly we found five different proteins which fit this criterion. Interesting among them were DCAF1 and COSP3 which are known interacting partners and were found to be highly ubiquitinated in control cells than in Cul4b deleted T cells. The exciting observation was that SMC1A is also differentially ubiquitinated (Figure 7H and Supplementary Figure 7H. While SMC1A was more ubiquitinated in control cells, it appears this ubiquitination is not targeting the protein for degradation. So, we posit that ubiquitination might be an important signal for ATM driven phosphorylation of SMC1A. The data has been discussed in the main manuscript and necessary changes were done in the results, discussion and material and methods and highlighted in yellow at lines 363-375, 503-510, 685-688, 934-947, 1116-1120.

3. In the beginning, Fig. 1, the authors show Cul4b is upregulated and becomes active after activation of both CD4 and CD8 T cells but for the rest of the study they chose to merely focus on CD4 T cells. Does Cul4b play a similar role in CD8 T cells?

Reviewer has correctly pointed out that Cul4b has similar impact on both CD4 and CD8 T cells. Our preliminary data on CD8 T cells have revealed that the defect may impact CD8 T cells even more than CD4 T cells. However, due to cell numbers and the need for extensive biochemistry to look at mechanisms, we have focused this manuscript on CD4 T cells. We plan to continue to look at CD8 T cells and plan to communicate these results in a separate manuscript in the future.

Reviewer #4: This manuscript by Dar et al. provided convincing evidence for a role of Cul4b in promoting T cell survival and expansion, and participating in DNA damage repair during T cell activation. In going forward, the authors may consider to address the following issues:

Major issues:

1. The authors claimed that in the absence of Cul4b, fewer cells entered the S and G2-M phase (Figure S5B-C) while more cells were undergoing apoptosis. It is not immediately obvious to this reviewer whether Cul4b-deficient cells had difficulty entering S phase compared with that of control. It would be helpful if Fig. S5C is presented with gated live cells so we can have a better picture of % of G1, S and G2/M between the two groups.

As suggested by the reviewer we did reanalyze the data and excluded the dead cells. The data clearly indicates that the Cul4b deficient has difficulty entering the G2-M phase. The reanalyzed data is included in the supplementary figure 5B-C and necessary changes are made in the text and highlighted in yellow at line 281-283, 1098-1101.

2. It is unclear what roles CUL4BDCAF1 plays in Smc1a phosphorylation. Is Smc1a ubiquitinated by Cul4b that facilitates ATM-dependent Smc1a phosphorylation? Are MRE11 and Rad50 ubiquitinated by Cul4b?

Reviewer has raised an excellent question that how Cul4b or DCAF1 regulate SMCA1A phosphorylation. To address this, we did carry out the diglycine remnant profiling to identify proteins that are less ubiquitinated in Cul4b-deficient CD4 T cells. We then

integrated this dataset with our IP MS/MS dataset and found five proteins that were identified in both datasets. Among these five proteins were DCAF1 and COSP3 which are known interacting partners of Cul4b, both were found to be more ubiquitinated in control cells than in Cul4b deleted T cells. Importantly, SMC1A is also more ubiquitinated in WT T cells (Figure 7H and Supplementary Figure 7H). While SMC1A was more ubiquitinated in control cells, this ubiquitination does not appear to be targeting SMC1a to the proteasome. We posit that optimal ubiquitination might be an important signal for ATM driven phosphorylation of SMC1A. The data has been included in the main manuscript and necessary changes were done in the results and material and methods and highlighted in yellow on lines 363-375, 503-510, 685-688, 934-947, 1116-1120..

3. Cul4b is on X chromosome. The Cul4bfl/fl should be changed to Cul4bfl/Y.

We have used both male and female mice in our invitro and *in vivo* experiments. So, to avoid ambiguity we referred it as Cul4bfl/fl. It has now been mentioned in material and methods that mice between 8 and 14 wk of age were used, and within experiments they were age, gender and cage matched (highlighted in yellow on lines 708-710)

Minor points:

1. Based on the results from cell transfer-induced colitis model and in vitro co-culture assay, CD4+ T cells require Cul4b to maintain survival and proliferation upon activation. it would be interesting to check under steady state, whether there is already altered/reduced CD4+ T cell number in colon of Cul4b deficient mice comparing to control mice, given that CD4+ T cells are mostly activated in intestine where large amount of commensal microbes colonize.

We agree with the reviewer that this would be an interesting experiment. However, this requires a thorough assessment of the types of microbes present in the GI tract and a full assessment of age gender and cages matched experiments. This is something we plan to look at in detail in future studies.

2. How about the CD4+ T cell subsets (Th17, Th2, Treg) in colon between Cul4b deficient and control mice?

We have done a thorough analysis of Th1, Th17 and Tregs in these mice at both steady state and in the colitis experiments. We have interesting findings regrading cytokine production and have evidence that this is distinct from the cell proliferation defect and involves a distinct mechanism. We plan to submit this information as a separate manuscript when we have collected sufficient data to support our hypothesis on how Cul4b regulates cytokine production.

3. In both colitis model and BM-chimera model, the in vivo proliferation (Ki-67, EdU)

and apoptosis (active Caspase-3, Annexin V) of CD4+ T cells from Cul4b deficient donor and control donor should be determined.

We thank reviewer for the comments, we now include Ki-67 data in the colitis and mixed chimera experiments (Figure 4E-F and Supplementary figure 4D-G respectively). The data clearly shows that higher percentage of control CD4 T cells are positive for Ki67 compared to Cul4b cKO cells. Again, validating that Cul4b is required for the proliferative capacity of CD4 T cells. The requisite changes are highlighted in yellow 216-221, 602-605, 239-241, 1078-1082

4. In Fig. 2B, migration is not a reliable criteria to indicate that CBF Cul4b is mostly neddylated. it is possible that the CBF Cul4b migrates differently on SDS-PAGE due to high salt buffer used in the extraction. The authors may consider to compare Cul4b with or without MLN4924 treatment.

As shown in the Figure 1a (last lane), We had already included the MLN4924 (NAEi) control to confirm that the upper band is the neddylated version of the protein. It should be noted that neddylation and de-neddylation are highly sensitive processes regulated in part by the COP9 signalosome. Inhibition of this complex is important to accurately analyze the neddylation of Cul4. Both buffer composition and incubation time greatly influences the extent of inhibition of the COP9 signalosome and thus neddylation of Cul4. This required significant optimization to allow an accurate analysis of the neddylated and de-neddylated versions of Cul4a and Cul4b (Supplementary Figure 1D). We have used low salt buffer for the extract of proteins ruling out the effect of high salt on the migration of the protein. We have added emphasis to the importance of buffers and conditions to the discussion of lysis conditions in the materials and methods section (line 830-834 highlighted in yellow).

5. In Fig. 6B-C, a critical control was missing: Comet assay should be carried out in control and Cul4b deficient cells in the absence of Etoposide treatment.

It is worthy to mention that only a fraction of the activated T cells will be undergoing sufficient DNA damage to show comet. We have done comet assay in the absence of the etoposide treatment, the amount of DNA in the tail was very low making it hard for the software to generate the reliable data. OpenComet plugin automatically identifies the comets and does not take into account the cells with more DNA in the tail or more DNA in the head. Automatically identifying these comets is currently a limitation of OpenComet but also helps to avoid the outliers.

6. In Fig. 7C-D, the authors should measure the half-life of DCAF1 post CHX treatment.

We thank reviewer for the comment, as suggested we did the CHX chase experiment and found that after 4hrs treatment a significant amount of DCAF1 is degraded. The

longer CHX treatment induced cell death in significant number of T cells making it difficult to get enough protein for the analysis. The data is included in the supplementary figure 6C and necessary changes are made in the text at line 328-331 1107-1110.

7. In Fig. S2B, CD3/CD28 or TCR stimulation increased CUL4b transcript. What about Cul4a?

We thank the reviewer for the comments. We believe that both Cul4a and Cu4b are the transcriptionally induced after TCR stimulation. Our RNA seq data showed that TCR stimulated CD4 T cells have moderately higher transcript levels of Cul4a than Cul4b (supplementary file 2). While our proteomics data confirmed that Cul4b is more abundant than Cul4a.

8. In the main text and figure legends, please change all "CD4 T cells" to "CD4+ T cells" to be consistent with the labels in figures. Also, the "Rag-/-" should be "Rag-/-".

We thank reviewer for the comment and necessary changes are done in the text and are highlighted in yellow

9. On page 5, 7th line to the last, it should be "… of total Cul4 proteins in CD4+ T cells" as the assay was conducted on CD4+ T cells but not total T cells.

We thank reviewer for the comment and necessary changes are done in the text and are highlighted in yellow

10. On page 5, 8th line to the last, it should be "(Figure 2C, Figure S2B)".

We thank reviewer for the comment and necessary changes are done in the text and are highlighted in yellow

Claesson, M. H., S. Bregenholt, K. Bonhagen, S. Thoma, P. Moller, M. J. Grusby, F. Leithauser, M. H. Nissen and J. Reimann (1999). "Colitis-inducing potency of CD4+ T cells in immunodeficient, adoptive hosts depends on their state of activation, IL-12 responsiveness, and CD45RB surface phenotype." J Immunol **162**(6): 3702-3710.
Leppkes, M., C. Becker, Ivanov, II, S. Hirth, S. Wirtz, C. Neufert, S. Pouly, A. J. Murphy, D. M. Valenzuela, G. D. Yancopoulos, B. Becher, D. R. Littman and M. F. Neurath (2009). "RORgamma-expressing Th17 cells induce murine chronic intestinal inflammation via redundant effects of IL-17A and IL-17F." Gastroenterology **136**(1): 257-267.
Mohammad, I., I. Starskaia, T. Nagy, J. Guo, E. Yatkin, K. Vaananen, W. T. Watford and Z. Chen (2018). "Estrogen receptor alpha contributes to T cell-mediated autoimmune inflammation by promoting T cell activation and proliferation." Sci Signal **11**(526).

Ostanin, D. V., J. Bao, I. Koboziev, L. Gray, S. A. Robinson-Jackson, M. Kosloski-Davidson, V. H. Price and M. B. Grisham (2009). "T cell transfer model of chronic colitis: concepts, considerations, and tricks of the trade." Am J Physiol Gastrointest Liver Physiol **296**(2): G135-146.
