# Supplementary material for: The E3 ubiquitin ligase Cul4b promotes CD4+ T cell expansion by aiding the repair of damaged DNA
Source: PLoS Biol. 2021 Feb 1;19(2):e3001041. doi: 10.1371/journal.pbio.3001041 (PMC7888682; doi:10.1371/journal.pbio.3001041)
Supplement: Attachment — Submitted filename: Reviewers Comments PlosB[2nd][29338].docx [file pbio.3001041.s021.docx]

**Reviewers’ comments**

**Rev. 1:**
The authors state in the rebuttal: "While analyzing for the possible substrate receptors (SRs) which could explain the phenotype, we took multiple things into consideration, for example concentration of substrate receptors, fold change in the concentration of the SRs between control and Cul4bcKO cells, and extent to which each substrate receptor was immunoprecipitated with Cul4b (based on peptide count)"

Reviewer response: It is important to note that CRL4 substrate receptor levels (or fold-change upon CUL4b loss) do not - in any way - imply that the most abundant receptor is responsible for the process studied. Even low-abundance receptor levels can drive essential cellular processes. The linkage from CUL4b to DCAF1 to SMC1a, working together "to allow survival and expansion of activated T cells" is possible, yet this remains somewhat tentative with the data presented. See also comments by reviewer #2.

**Response:** We thank reviewer for the comment and acknowledge that low abundance substrate receptors can be important and might be helping CRL4B to regulate multiple processes in T cells. However, we would like to mention that under invitro conditions we were able to detect only four substrate receptors that interacted with Cul4b. Among them DCAF1 was more frequently identified compared to DCAF7 (1 peptide), DCAF8 (2 peptides) and DCAF3 (10 peptides). Given that DCAF1 is required for T-cell expansion and function during anti-viral and autoimmune responses in vivo (doi.org/10.1038/ncomms10307) and that DCAF1 has been shown to regulate Treg senescence (DOI:[10.1172/JCI136466](https://doi.org/10.1172/JCI136466)) we provide evidence that that it does this via interactions with Cul4b. Supporting this, deletion of Cul4b in T cells resulted similar phenotype in CD4 T cells. Thus, we posit that Cul4b and DCAF1 work together to regulate the DNA damage pathway to aid T cell proliferation and survival. Based on these data, we believe that DCAF1 might be a key substrate receptor under these conditions. We have omitted the sentence “major substrate receptor” and replaced it with “key substrate receptor” (line 337). Despite this we do not rule out the possibility of other substrate receptors helping CRL4 complex in regulating diverse functions and have added wording to this effect into the discussion (472-474).

The authors state in the rebuttal: "Both buffer composition and incubation time greatly influences the extent of inhibition of the COP9 signalosome and thus neddylation of Cul4."

Reviewer response: I am not aware of this being formally shown in the literature. What is this statement based on? The CSN5 subunit of the signalosome is a Zinc protease, so Zn-chelators in the buffer could play a role in modifying activity. Good inhibitors are at hand for CSN though and commercially available (see <https://www.nature.com/articles/ncomms13166>). Could this comment possibly be an indication that the experimental read-outs are not very robust? See my previous comments (repeated below).

**Response:** We thank reviewer for this comment. Our lysis buffer contains a known Zn-Chelator (1’10 orthphenarthroline (OPA) (line 833-834). However, even under conditions in which we use a higher concentration of OPA, the deneddylation was not completely blocked (as seen in the inputs of our IP western blots). Based on our data, we believe that the method we used to detect neddylation of Cul4b and Cul4a in Figure 1 is robust and prevents any artifacts induced during lysis and incomplete inhibition of CSN5 of COP9 signalosome.

Few studies have focused on the neddylated and un-neddylated forms of the proteins, and in those that do, it is not discussed in much detail. Yong chao Zhao et al doi: [10.1089/ars.2013.5795](https://dx.doi.org/10.1089%2Fars.2013.5795) using MLN4924 have shown both Nedd8 and non-Nedd8 versions of the Cullins. In another study by [Boon Kim Boh](https://www-sciencedirect-com.proxy.library.upenn.edu/science/article/pii/S0022283611002889?via%3Dihub#!) et al, ([doi.org/10.1016/j.jmb.2011.03.023](https://doi-org.proxy.library.upenn.edu/10.1016/j.jmb.2011.03.023) ) disparity in the forms of the neddylated and non-neddylated version can be seen in Fig 1b and Fig 2a. Figure 1b shows equal proportions of the neddylated and non-neddylated form (lane 2) which decreases after MLN4924 treatment. While in Figure 2b, almost all Cul2 protein is shown to be in the neddylated form. Further, Jessica S. Brown et al (doi.org/10.1016/j.celrep.2015.03.058) in Fig 3C ( IP western inputs) have shown single band for both Cul4b and Cul4a. Although these disparities do not change the outcome of either the published studies or our current studies, the variation might be to differences in assay conditions or the types of cells selected for the studies.

Previous comment: "Figure 7G: IP westerns are not too convincing. For example, how reproducible is the co- immunoprecipitation of Smc1a? Smc1a seems hard to detect, but appears to be almost equally abundant in Suppl. Fig. 6E in the IgG control. Why are there two bands for DCAF1 in Fig. 7G, but only one in Figure 7B and C and Suppl. Fig. 6E? Can the authors be sure that the bands detected by their antibodies in Fig. 7G and Suppl. Fig. 6E indeed correspond to the appropriate target protein?"

While this has been partially addressed by IP-MS for SMC1, which is appreciated, the identity/variability of the two vs. one band for DCAF1 remains a concern.

**Response:** We thank reviewer for appreciating the new data added to the manuscript. With regard to the DCAF1, we would like assure the reviewer that during past two months we have made significant progress with regards to identifying these two bands. A similar band pattern for DCAF1 was observed in the study on DCAF1 and T cells (Supplementary Figure 7 raw blots) (doi.org/10.1038/ncomms10307). However, in this study they have not discussed this phenomenon as they focussed on the data where cells were stimulated for 24hr. We have now used 3 different antibodies specific for different regions of the protein and we are now convinced that these bands both represent DCAF1. Additionally, we have observed this band in both mouse and human (Jurkat) T cells (after different treatments). We have also determined that the intracellular localization of the full length and processed (faster migrating) band is different. These are findings we plan to publish separately as this will require a good deal more characterization and validation.

Rev. 2:
The resubmitted manuscript by Dar et al. has been substantially improved and has addressed most of the comments and concerns raised by the reviewers.

There remains some concern over the interpretation of the results and potential confounding variables, as well as a few points of clarification. These and other concerns are described further below.

Major concerns:
1. The authors justify not analyzing T cell repertoire by stating that "The rearrangement of TCR genes takes place before T cells enter the DP stage prior to Cul4b deletion." That is factually incorrect. TCR alpha chain rearrangement occurs during the DP stage. Thus, the original concern that the T cell repertoire may be significantly altered in the Cul4b conditional knockout mice, given evidence that loss of DCAF1 in B cells or T cells alters the immune repertoire (e.g. in B cells by dysregulating RAG1 levels and in T cells possibly interfering with DNA repair), still stands. This may or may not be masked by redundancy with Cul4a. This would also have implications for understanding the outcome of the adoptive transfer experiments in RAG1-/- mice, particularly if the TCR repertoire was severely restricted. TCR spectratyping analysis is quite feasible now and should answer the question.

Response: We acknowledge the reviewers concern regarding analysing the T cell repertoire. We don’t rule out the role of Cul4b in TCR gene rearrangement. However, this manuscript is focused on the role of Cul4b in the peripheral expansion of T cells. It is important to note that the phenotype we observe is unlikely to be impacted significantly by a skewing of T cell repertoire. Supporting this, when we assess the WT and Cul4b-deficient T cells in mixed chimeras, there is very little difference in the frequencies of naïve CD4+ T cells. In contrast, we have found that Cul4b deletion in T cells impacts their proliferation and survival when isolated as naïve T cells and stimulated in vitro with anti-CD3 and anti-CD28. It would difficult to imagine that this is due to a skewing of the TCR repertoire.
While we appreciate that it will be interesting to assess the role of Cul4b in TCR repertoires, we believe that adding data on T cell repertoires is unlikely to add to the central theme of the manuscript.

2. The inclusion of the ratio of neddylated and non-neddylated forms of Cul4b in Fig. 1 confirms the suspicion that this ratio doesn't change much in response to TCR stimulation (none of the changes appears statistically significant). Thus, while the level of Cul4b increases robustly and is not in question, the activation of Cul4b is not necessarily enhanced. Thus, the authors should revise the some of the findings in the text and summary. For example, in the summary it states "Cul4b levels and activity increase following TCR stimulation", but the latter claim is not really supported by the analysis of Cul4b neddylation.

**Response:** The reviewer is correct. As suggested by the reviewer we have modified the statement in the results section of manuscript (Line 99) as well as in the abstract (Line 32).

3. While the inclusion of additional data suggesting an association between Cul4b and SMC1a and MRE11A, and the reduction of SMC1a phosphorylation upon loss of Cul4b, is gratifying and improves rigor, the mechanistic link is at best only correlative. However, it is unclear what phospho-specific antibody is being used to detect SMC1. The antibody used is listed as being from Abcam, but there are several available for different sites: S360, S957, and S966. The specific product number is not included. The question is raised as to whether the phospho-site they are investigating makes sense with respect to TCR signaling, and loss of phosphorylation is a general phenomenon, or is site-specific. There is no justification provided for the choice of phospho-specific SMC1 antibody. This should be included at least, and would be more convincing if it were observed with another antibody as well (there are some available from CST which are better characterized).

**Response:** SMC1A is phosphorylated on Serine 957 and 966 by protein kinase ataxia-telangiectasia mutated (ATM) following DNA damage an event thought to be a downstream effector of ATM-NBSI-BRCA pathway that affects cell survival and chromosomal stability maintenance after DNA damage. The phosphorylated form on Ser-957 and Ser-966 is also known to associate with chromatin during G1/S/G2 phases. Given that, following TCR stimulation, ATM activity increases (Fig 5) we hypothesized that Serine 957 and 966 might be the targets. Rather than focusing on both, we used an antibody specific for Serine 957. We made the necessary changes in the text to mention the rationale of using serine 957 antibody and mentioned that the serine 957 specific antibody was chosen. These changes can be found in the lines 382-388 and 844.

4. In the previous version of the manuscript, the primary data for the mass spectrometry results was unavailable. It is interesting to note that the Cul4b IP, but not the VprBP/DCAF1 IP, pulls down another E3 ubiquitin ligase, Rbbp6, with as much confidence as other factors listed in Fig. 7F (12 peptides). Why this is noteworthy is that Miotto et al. published in Cell Reports (2014 Apr 24;7(2):575-587) that this E3 ligase plays a role in DNA replication and that its loss lead to increased spontaneous DNA damage, similar to the phenotype that is observed in the Cul4b-deficient cells. It is unclear why the identification of this protein was not noted in Fig. 7F. What the Miotto paper suggests is the possibility that Cul4b has a mechanistic link to Rbbp6. This should at least be acknowledged.

Response: We thank reviewer for this suggestion. The study published by Miotto et al. have shown that cells lacking RBBP6 experience reduced replication fork progression and increased damage at common fragile sites due to ZBTB38 accumulation and MCM10 downregulation. On contrary we have found that MCM10 accumulates in Cul4b deleted T cells by around 1.5 log2 fold (proteomics data). So, these two ligases might be working through different mechanisms. However, as suggested by the reviewer we have added this protein to the list of proteins associated with DNA damage response as well. Necessary changes have been done in the manuscript to acknowledge the work of Miotto et al.

5. In re-reviewing the data in Fig. 6, it is not clear whether the authors can really attribute a role for Cul4b in the DNA damage response, because basal levels of activation of DDR factors are increased during activation, as the authors point out in line 294 "…Cul4b promotes the DNA damage response (DDR) in activated CD4+ T cells at a time point that correlates with DNA synthesis and proliferation". However, an alternative and nuanced interpretation is that due to Cul4b-dependent defects in DNA replication, spontaneous DNA damage increases, thus activating the DDR. Though levels of DDR factor staining in Fig. 6B increase upon etoposide treatment, it is not clear whether this is simply additive to (or significantly enhanced over) the effects observed by T cell stimulation. It is therefore felt that attributing a unique role of Cul4b in activating the DDR is not strongly supported by the evidence, and the text and title should be revised to reflect that possibility.

**Response:** It is important to note that these processes are linked and Cul4b might be at the crossroad of both events. Cul4b is known to regulate the refiring of origins of replication by targeting the CDT1 degradation and hence DNA damage. So, we don’t rule out that there may be increased DNA damage due to deletion of Cul4b. This is discussed at line 517-521 in the discussion section. Apart from the increased DNA damage, the DNA repair response is also altered as evident by decreased SMC1a phosphorylation required for DNA repair. Knowing SMC1a is target of pATM, which is significantly higher in the cul4b deleted T cells (figure 6F-G), the concomitant increase in SMC1A phosphorylation (Figure 7J) was not observed and was even less than that observed in control CD4 T cells.

Further, looking at the data in Figure 6B and Figure 6D-K, the DDR factors are significantly increased over TCR stimulation compared to the unstimulated T cells. These factors are further increased after etoposide treatment clearly suggesting that is additive to the effects observed only with TCR stimulation.

Minor concerns:
1. The authors should indicate how many technical replicates were performed for the mass spectrometry experiments (e.g. Fig. 7F).

The mass spectrometry data in fig 7F is from a single representative and the relevant proteins were validated by IP Western blots. The necessary changes have been done in the manuscript at line 685.
