# Supplementary material for: The E3 ubiquitin ligase Cul4b promotes CD4+ T cell expansion by aiding the repair of damaged DNA
Source: PLoS Biol. 2021 Feb 1;19(2):e3001041. doi: 10.1371/journal.pbio.3001041 (PMC7888682; doi:10.1371/journal.pbio.3001041)
Supplement: Attachment — Submitted filename: reviewers comments 11221.docx [file pbio.3001041.s022.docx]

1. Please provide a legend for your S7 Data file at the end of the manuscript.

**The legend to the S7 data file is placed at the end of the manuscript.**

2. Please rename your figure files so that they are labeled as, for instance, "Fig.tif", and not "Fig1.tif.tif".

**Figures have been renamed as Fig.tif**
